# Identification of lipid synthesis genes in *Schizochytrium* sp. and their application in improving eicosapentaenoic acid synthesis in *Yarrowia lipolytica*

**DOI:** 10.1186/s13068-024-02471-y

**Published:** 2024-02-24

**Authors:** Yu-Lei Jia, Qing-Ming Zhang, Fei Du, Wen-Qian Yang, Zi-Xu Zhang, Ying-Shuang Xu, Wang Ma, Xiao-Man Sun, He Huang

**Affiliations:** School of Food Science and Pharmaceutical Engineering, Nanjing Normal University, Nanjing, 210000 China

**Keywords:** Eicosapentaenoic acid, Lipid, *DGAT*, *Yarrowia lipolytica*, Lipidomic analysis, Triglyceride

## Abstract

**Background:**

Eicosapentaenoic acid (EPA) is widely used in the functional food and nutraceutical industries due to its important benefits to human health. Oleaginous microorganisms are considered a promising alternative resource for the production of EPA lipids. However, the storage of EPA in triglyceride (TG) becomes a key factor limiting its level.

**Results:**

This study aimed to incorporate more EPA into TG storage through metabolic engineering. Firstly, key enzymes for TG synthesis, the diacylglycerol acyltransferase (*DGAT*) and glycerol-3-phosphate acyltransferase (*GPAT*) genes from *Schizochytrium* sp. HX-308 were expressed in *Yarrowia lipolytica* to enhance lipid and EPA accumulation. In addition, engineering the enzyme activity of *DGAT*s through protein engineering was found to be effective in enhancing lipid synthesis by replacing the conserved motifs “HFS” in *ScDGAT2A* and “FFG” in *ScDGAT2B* with the motif “YFP”. Notably, combined with lipidomic analysis, the expression of *ScDGAT2C* and *GPAT2* enhanced the storage of EPA in TG. Finally, the accumulation of lipid and EPA was further promoted by identifying and continuing to introduce the *ScACC*, *ScACS*, *ScPDC*, and *ScG6PD* genes from *Schizochytrium* sp., and the lipid and EPA titer of the final engineered strain reached 2.25 ± 0.03 g/L and 266.44 ± 5.74 mg/L, respectively, which increased by 174.39% (0.82 ± 0.02 g/L) and 282.27% (69.70 ± 0.80 mg/L) compared to the initial strain, respectively.

**Conclusion:**

This study shows that the expression of lipid synthesis genes from *Schizochytrium* sp. in *Y. lipolytica* effectively improves the synthesis of lipids and EPA, which provided a promising target for EPA-enriched microbial oil production.

**Supplementary Information:**

The online version contains supplementary material available at 10.1186/s13068-024-02471-y.

## Background

According to reports, the market for omega-3 fatty acids will be expanding at the compound annual growth rate of 7.7% by 2027 [1]. Eicosapentaenoic acid (EPA, C20:5Δ^5, 8, 11, 14, 17^), as an important long-chain ω- 3 polyunsaturated fatty acids (PUFAs), has been proven to have significant contributions to human health, thus occupying a place in the functional food and nutraceutical industry [2]. Currently, high-quality EPA is mainly extracted from deep-sea fish oil. However, the unsustainability of deep-sea fish resources and the severe damage to marine ecosystems due to overfishing have limited the production and sale of EPA [2]. In addition, the enormous gap between global EPA production and recommended dietary intake indicates the need to develop a sustainable and safe method for producing EPA [3]. Oleaginous microorganisms are considered a promising alternative resource for the production of EPA lipids because they can accumulate lipids exceeding 20% of their dry cell weight (DCW) [4]. In addition, they have several advantages such as ease of culture, multiple lipid composition, and extensive carbon source substrate utilization [5].

Triglyceride (TG) is the major storage lipid in oleaginous cells, which is formed mainly through the Kennedy pathway and exists in both the endoplasmic reticulum and the chloroplasts (Additional file 1: Fig. S1) [6]. Diacylglycerol acyltransferase (*DGAT*) is considered a rate-limiting and key enzyme that catalyzes the final acylation of diacylglycerol (DG) to form TG [7]. Previous studies have shown when *CrDGTT1* from *Chromochloris zofingiensis* was expressed in *Nannochloropsis oceanica*, the percentage of EPA in TG and the content of TG-derived EPA increased by 4.8-fold and 10.1-fold, respectively, compared to the control strain [8]. There are three distinct types of *DGAT*s related to TG biosynthesis, including *DGAT* 1, 2, and 3. *DGAT*1 and *DGAT*2 are membrane-binding protein and *DGAT*3 is soluble cytosolic enzyme [9]. Although they catalyze the same enzymatic reaction, there is no apparent homology in the amino acid sequence between *DGAT1* and *DGAT2*. Modification of *DGAT* genes has already been attempted and is considered to be the best strategy for manipulating TG accumulation in microorganisms [10]. *DGAT2* has been identified as a more efficient TG biosynthetic enzyme than *DGAT1*, especially for unusual fatty acids, and has been transferred to various cells to produce and store large amounts of fatty acids [11]. The functions of four *DGAT2*s (*DGAT2A*, *DGAT2B*, *DGAT2C*, and *DGAT2D*) in *Aurantiochytrium* sp. SD116 were investigated, and it was found that *DGAT2C* was mainly responsible for connecting PUFAs to the sn-3 position of the TG molecules, while *DGAT2A* and *DGAT2D* mediated the SFA-TGs production [5]. Overexpression of *DGAT2* in *Nannochloropsis oceanica* resulted in a 69% increase in TG content. In addition, SFAs and PUFAs were found to be increased whereas monounsaturated fatty acids content decreased [12]. In another study, overexpression of *DGAT2* in *Chlamydomonas reinhardtii* resulted in a ninefold increase in TG content [13]. Therefore, *DGAT* is considered a key target for manipulating TG synthesis, and a better understanding of *DGAT* properties may provide new possibilities for the enrichment of required fatty acids in TG.

*Schizochytrium* sp., as a marine heterotrophic microorganism, can synthesize and accumulate large amounts of TG and is rich in PUFAs [14]. Under optimized conditions, the lipid content of some *Schizochytrium* strains can reach over 55% of DCW [15]. Therefore, the exploration of its TG and lipid synthesis pathway genes is of great research significance. In this study, *Y. lipolytica* was used as a chassis cell into which the TG and lipid synthesis genes from *Schizochytrium* sp. HX-308 were introduced to enrich EPA in its TG for the first time through a “push–pull-promote” metabolic engineering strategy. Meanwhile, we combined lipidomics techniques with protein engineering to explore their role in mediating EPA in TG assembly. This study aims to provide a valuable reference for the improvement of EPA biosynthesis.

## Results and discussion

### Bioinformatic analysis of *DGAT*s

Four putative DGAT genes, *ScDGAT2A* (gene ID: A6553), *ScDGAT2B* (gene ID: A7082), *ScDGAT2C* (gene ID: A7100), and *ScDGAT3* (gene ID: A7950), were identified based on functional annotation of the genome of *Schizochytrium* sp. HX-308. ID: A7950). Based on the phylogenetic analysis of amino acid sequences, *ScDGAT2A*, *ScDGAT2B*, and *ScDGAT2C* were classified as *DGAT2* family, while *ScDGAT3* was classified as *DGAT3* family (Additional file 1: Fig. S2a). It has been reported that the *DGAT3* clade shares a most recent ancestor with a group of uncharacterized proteins from cyanobacteria, suggesting a prokaryotic origin, whereas the canonical *DGAT*s, *DGAT1* and *DGAT2*, are only related to proteins from eukaryotes [16]. In addition, most microalgae contain one or more *DGAT* genes, which may be closely related to their high-level storage of PUFAs. According to reports, the DHA content of *Schizochytrium* sp. can reach over 35% of total fatty acids [17].

Multiple-sequence alignment of *Schizochytrium* sp. HX-308 *DGAT*s was performed with other *DGAT* amino acid sequences (Additional file 1: Fig. S2b). *ScDGAT2A*, *ScDGAT2B*, and *ScDGAT2C* were found to contain six highly conserved motifs that were identified as signature motifs within the DGAT2 subfamily, namely Motif 1 (PH Block), Motif 2 (PR Block), Motif 3 (GGE Block), Motif 4 (RGFA Block), Motif 5 (VPFG Block), and Motif 6 (G Block) [18]. The full length of *ScDGAT2A*, *ScDGAT2B*, *ScDGAT2C*, and *ScDGAT3* was 936, 857, 466, and 632 amino acid residues, respectively. Furthermore, in addition to *ScDGAT3*, *ScDGAT2A*, *ScDGAT2B*, and *ScDGAT2C* contain 11, 11, and 3 distinct transmembrane domains, respectively (Additional file 1: Fig. S3), which is consistent with previous reports that *DGAT2* is membrane-binding protein while *DGAT3* does not have transmembrane structure [16].

Subcellular localization prediction of the proteins showed that all of *ScDGAT2A*, *ScDGAT2B*, *ScDGAT2C*, and *ScDGAT3* are localized in the endoplasmic and participate in the conventional pathway of TG synthesis here (Additional file 1: Fig. S4) [19]. In addition, *ScDGAT3* was also found to be localized in chloroplasts. According to reports, most of the algal sequences within the *DGAT3* group have predicted chloroplast localization [20], indicating that they may have the ability to accumulate light-dependent TG [16]. However, *Schizochytrium* sp. is a type of marine microalgae that lacks chloroplasts. Therefore, further experimental verification on this speculation is needed. The conservative domain prediction showed that *ScDGAT2A*, *ScDGAT2B*, and *ScDGAT2C* include a lysophospholipid acyltransferase (LPLAT) superfamily domain, which may be involved in the acyltransferase activity (Additional file 1: Fig. S5). While *ScDGAT3* includes the abhydrolase superfamily domain, which belongs to the α/β hydrolase superfamily that contains proteases, lipases, peroxidases, esterases, epoxide hydrolases and dehalogenases [21].

### Effects of *DGAT*s on lipid and fatty acid profile

**Fig. 1:**
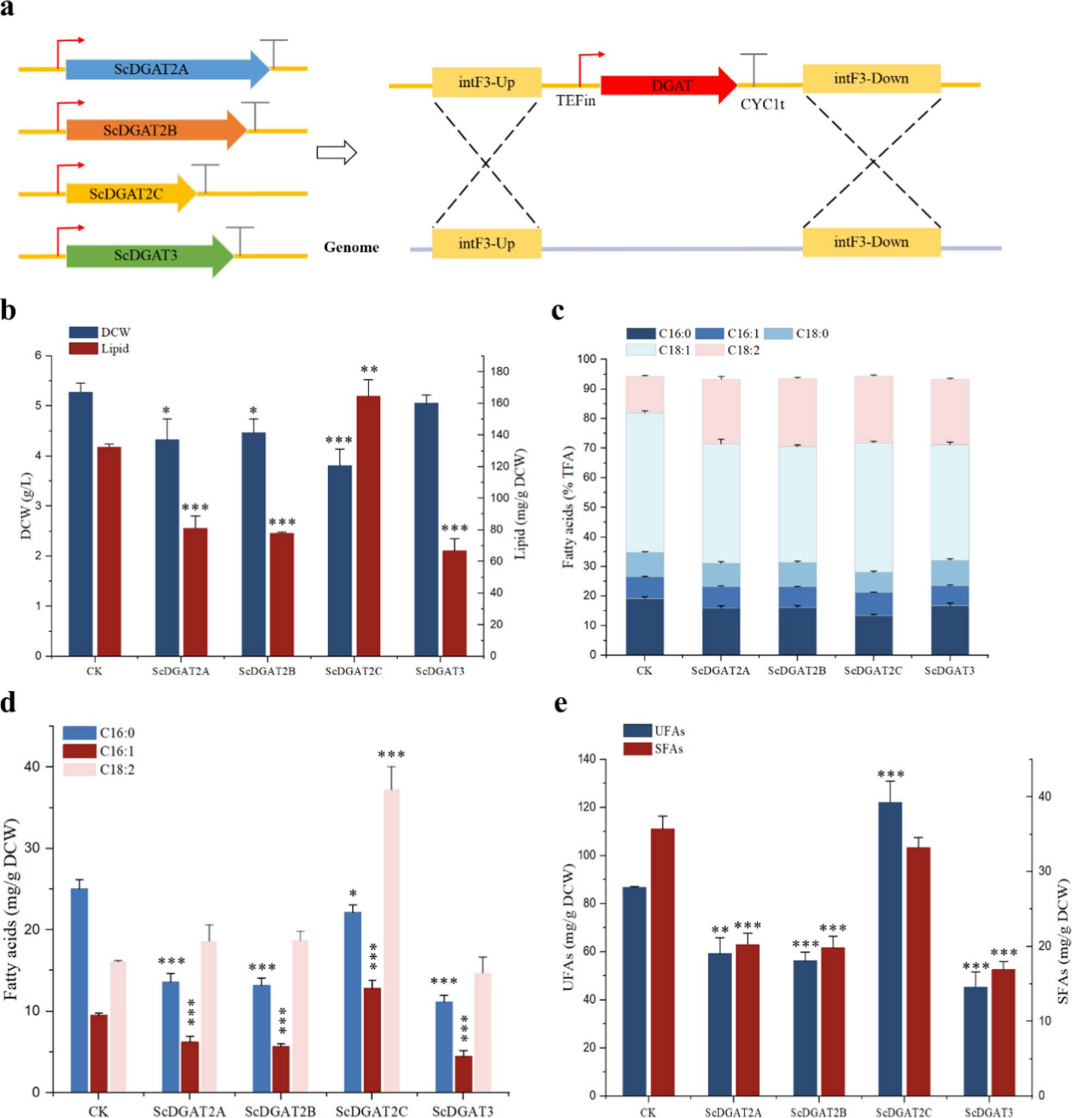
Effects of *DGAT*s on lipid and fatty acid synthesis in *Y. lipolytica* mutant strain (CK). **a** Scheme of the expression of *DGAT* genes controlled by the TEFin promoter and CYC1t terminator at the intF3 integration site based on homologous recombination. intF3-Up, upstream homologous arm of integration site intF3; intF3-Down, downstream homologous arm of integration site intF3. Effects of *DGAT*s on lipid and DCW(**b**), fatty acid composition (**c**), C16:0, C16:1, and C18:2 content (**d**), and UFAs and SFAs content (**e**) in *Y. lipolytica* mutant strain (CK). Three biological replicates were used and mean values ± SD (n = 3) are shown. Two-way ANOVA with Tukey’s multiple comparisons test. *P < 0.05, **P < 0.01, and ***p < 0.001 compared with the CK

In order to characterize the functions of four *DGAT* genes, yeast expression vectors for these genes were constructed and integrated into the intF3 loci of the *Y. lipolytica* mutant strain (CK) genome (Fig. 1a), in which *DGA1*, *DGA2*, and *LRO1* that catalyze TG formation were all knocked out, resulting in fatty acids that can only exist in free form [22]. In addition, the intF3 loci were selected based on previous reports that they can be used to integrate heterologous genes without affecting cell growth [23]. Subsequently, the genomic DNA of *Y. lipolytica* was extracted and used as a template for PCR amplification, and electrophoretic bands of the target size were successfully obtained (Additional file 1: Fig. S6a), indicating that these *DGAT* genes had been successfully integrated into the genome of *Y. lipolytica* and the engineered strains ScDGAT2A, ScDGAT2B, ScDGAT2C, and ScDGAT3 were obtained.

In this work, the expression of *ScDGAT2A*, *ScDGAT2B*, and *ScDGAT2C* resulted in a 21.94%, 18.12%, and 38.58% decrease in DCW content compared to the CK strain, respectively, except for the DCW of the ScDGAT3 engineered strain, which was not significantly changed compared to the CK strain (Fig. 1b). In addition, compared to the CK strain, the expression of *ScDGAT2A*, *ScDGAT2B*, and *ScDGAT3* resulted in a 63.26%, 70.14%, and 98.59% decrease in lipid content, respectively (Fig. 1b). In contrast, the expression of *ScDGAT2C* resulted in a 24.25% increase in lipid content compared to the CK strain (Fig. 1b), indicating that *ScDGAT2C* is closely associated with lipid synthesis, which is similar to the results reported previously. Expression of *DGAT* from *Arabidopsis thaliana* in *Nannochoropsis oceanica* resulted in an increase in total lipid content from 18.72% of wild-type strain to 24.49% [24]. In another study, overexpression of *ACC1* and *DGA1* in *Y. lipolytica*, the lipid content of the engineered strain increased from 8.7% to 41%. Moreover, the lipid content of the engineered strain reached 61.7% after 120 h cultivation [25].

Subsequently, the fatty acid profiles of CK and the four engineered strains were analyzed. The expression of all four *DGAT* genes resulted in significant changes in the fatty acid profiles (Fig. 1c), which is consistent with the results previously reported in the overexpression of *DGAT* gene in *Thalassiosira pseudonana* and *Phaeodactylum tricornutum* [26, 27]. In this work, the significant changes in the fatty acid profiles were C16:0 and C18:2. The C16:0 ratio in total fatty acids of ScDGAT2A, ScDGAT2B, ScDGAT2C, and ScDGAT3 strain decreased from 19.23 ± 0.55% of CK strain to 16.02 ± 0.76%, 16.16 ± 0.60%, 13.46 ± 0.43%, and 16.83 ± 0.80%, respectively. In contrast, the C18:2 ratio in total fatty acids of ScDGAT2A, ScDGAT2B, ScDGAT2C, and ScDGAT3 strain increased from 12.38 ± 0.26% of CK strain to 21.83 ± 0.99%, 23.02 ± 0.40%, 22.59 ± 0.37%, and 22.06 ± 0.30%, respectively. Compared to the CK strain, the C16:1 content was decreased in the ScDGAT2A, ScDGAT2B, and ScDGAT3C strains, whereas increased in the ScDGAT2C strain. In addition, the C18:2 content in the ScDGAT2C strain increased by 131.18% compared to the CK strain (Fig. 1d), indicating that the expression of ScDGAT2C enhanced the accumulation of C18:2 in TG.

Compared to the CK strain, the UFAs and SFAs contents in ScDGAT2A, ScDGAT2B, and ScDGAT3 strains were significantly reduced (Fig. 1e). Although SFAs content in ScDGAT2C strain was also reduced, the UFAs content was significantly increased by 40.52% compared to the CK strain (Fig. 1e), which was similar to the results previously reported [24]. These results suggest that the expression of *ScDGAT2C* improved the assembly of UFAs-TG in *Y. lipolytica*.

### Engineering the enzyme activity of *DGAT*s

**Fig. 2:**
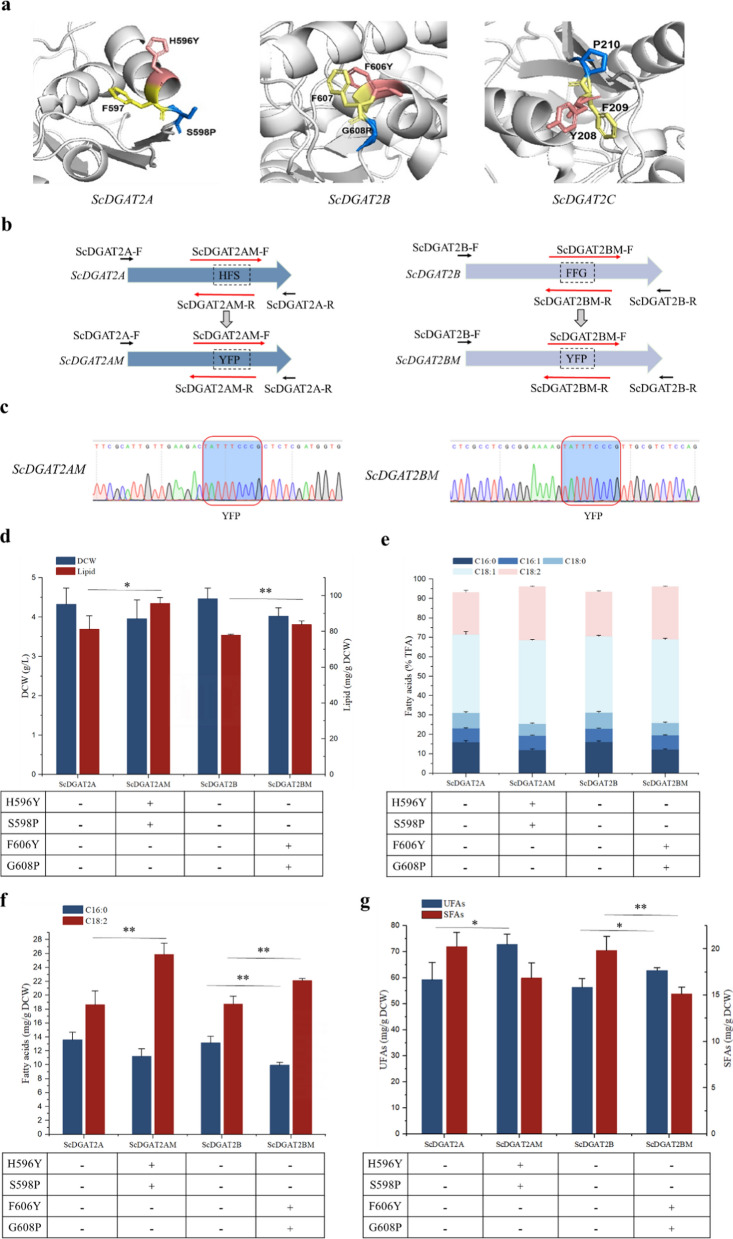
Effect of site-directed mutation of *DGAT*s on lipid and EPA synthesis. (**a**) The region of site-directed mutation in *ScDGAT2A* and *ScDGAT2B*. (**b**) Schematic diagram of site-directed mutation of *ScDGAT2A* and *ScDGAT2B*. (**c**) Sanger sequencing confirmation of site-directed mutation of *ScDGAT2A* and *ScDGAT2B*. Effects of site-directed mutation of *ScDGAT2A* and *ScDGAT2B* on lipid and DCW (**d**), fatty acid composition (**e**), C16:0 and C18:2 content (**f**), and UFAs and SFAs content (**g**) in *Y. lipolytica* mutant strain (CK). Three biological replicates were used and mean values ± SD (n = 3) are shown. Student’s t test was used for statistical analysis, and statistical significance is indicated as *P < 0.05, **P < 0.01, and ***p < 0.001

Previous studies have shown that several conserved amino acid motifs are essential for the high-level enzyme activity of *DGAT*, such as the “YFP” and “PH” motif in yeast *DGAT2*, which are also conserved in animals and higher plants [28]. The replacement of “YFP” to “AAA” and His^193^ to Ala^193^ in *Saccharomyces cerevisiae* has been reported to result in an almost complete loss of enzyme activity [28]. Protein sequence alignment revealed that only *ScDGAT2C* protein had a complete “YFP” motif, while the *ScDGAT2A* and *ScDGAT2B* proteins had “HFS” and “FFG” motifs, respectively (Additional file 1: Fig. S2b, Fig. 2a), which may be the reason why enzyme activity of *ScDGAT2C* was higher than *ScDGAT2A* and *ScDGAT2B*. Therefore, we boldly speculate that replacing the “HFS” motif in *ScDGAT2A* and the “FFG” motif in *ScDGAT2B* with the “YFP” motif through site-directed mutation may be able to enhance the enzyme activity to some extent and thus improve lipid accumulation (Fig. 2a). Then, using the *ScDGAT2A* gene as a template, primer pairs ScDGAT2A-F/ScDGAT2AM-R and ScDGAT2AM-F/ScDGAT2A-R were used to amplify and obtain two fragments with the size of 1814 bp and 1057 bp, respectively, and the *ScDGAT2AM* gene was subsequently obtained by homologous recombination of the two fragments (Fig. 2b). Similarly, Using the *ScDGAT2B* gene as a template, primer pairs ScDGAT2B-F/ScDGAT2BM-R and ScDGAT2BM-F/ScDGAT2B-R were used to amplify and obtain two fragments with the size of 1855 bp and 790 bp, respectively, and then the *ScDGAT2BM* gene was obtained by homologous recombination of the two fragments (Fig. 2b). Gene sequencing results show that the “HFS” motif in ScDGAT2A and the “FFG” motif in *ScDGAT2B* have been successfully replaced with the “YFP” motif (Fig. 2c). Subsequently, the *ScDGAT2AM* and *ScDGAT2B*M genes were integrated into the intF3 loci of the *Y. lipolytica* mutant (CK) strain genome in the same manner as described above and the ScDGAT2AM and ScDGAT2BM strains were obtained.

There was no significant change in DCW of ScDGAT2AM and ScDGAT2BM strains compared to the ScDGAT2A and ScDGAT2B strains, while lipid content was significantly increased (Fig. 2d). Compared to the ScDGAT2A and ScDGAT2B strains, the lipid content of ScDGAT2AM and ScDGAT2BM strains increased by 17.80% and 7.61%, respectively. In addition, there were significant changes in the fatty acid profiles (Fig. 2e). The C16:0 and C18:0 ratios in total fatty acids of ScDGAT2AM strain decreased from 16.02 ± 0.76% and 7.83 ± 0.58% of ScDGAT2A strain to 12.01 ± 0.52% and 6.05 ± 0.26%, respectively, while C18:1 and C18:2 ratios in total fatty acids increased from 40.34 ± 1.56% and 21.83 ± 0.99% of ScDGAT2A strain to 42.90 ± 0.38% and 27.76 ± 0.17%, respectively. The C16:0 and C18:0 ratios in total fatty acids of ScDGAT2BM strain decreased from 16.16 ± 0.60% and 8.20 ± 0.44% of ScDGAT2B strain to 12.25 ± 0.28% and 6.41 ± 0.35%, respectively, while C18:1 and C18:2 ratios in total fatty acids increased from 39.23 ± 0.50% and 23.02 ± 0.40% of ScDGAT2B strain to 42.87 ± 0.64% and 27.30 ± 0.19%, respectively. In addition, the C16:0 content of ScDGAT2AM strain showed no significant difference compared to the ScDGAT2A strain, while the C18:2 content increased by 39.07% compared to the ScDGAT2A strain (Fig. 2f). Compared to the ScDGAT2B strain, the C16:0 content of ScDGAT2BM strain decreased by 32.53%, while the C18:2 content increased by 18.04% (Fig. 2f). These results indicate that replacing the “HFS” motif in *ScDGAT2A* and the “FFG” motif in *ScDGAT2B* with the “YFP” motif can enhance the accumulation of C18:2 in TG.

Compared to the ScDGAT2A strain, the SFAs content of ScDGAT2AM strain showed no significant change, while the UFAs content was significantly increased by 22.83%. Compared to the ScDGAT2B strain, the ScDGAT2BM strain showed a significant decrease in SFAs content by 31.13%, while the UFAs content was significantly increased by 11.55% (Fig. 2g). These results indicate that the replacement of the “HFS” motif in *ScDGAT2A* and the “FFG” motif in *ScDGAT2B* with the “YFP” motif enhanced the assembly of UFAs-TG in *Y. lipolytica*.

### Effects of *DGAT*s on lipid and EPA accumulation

**Fig. 3:**
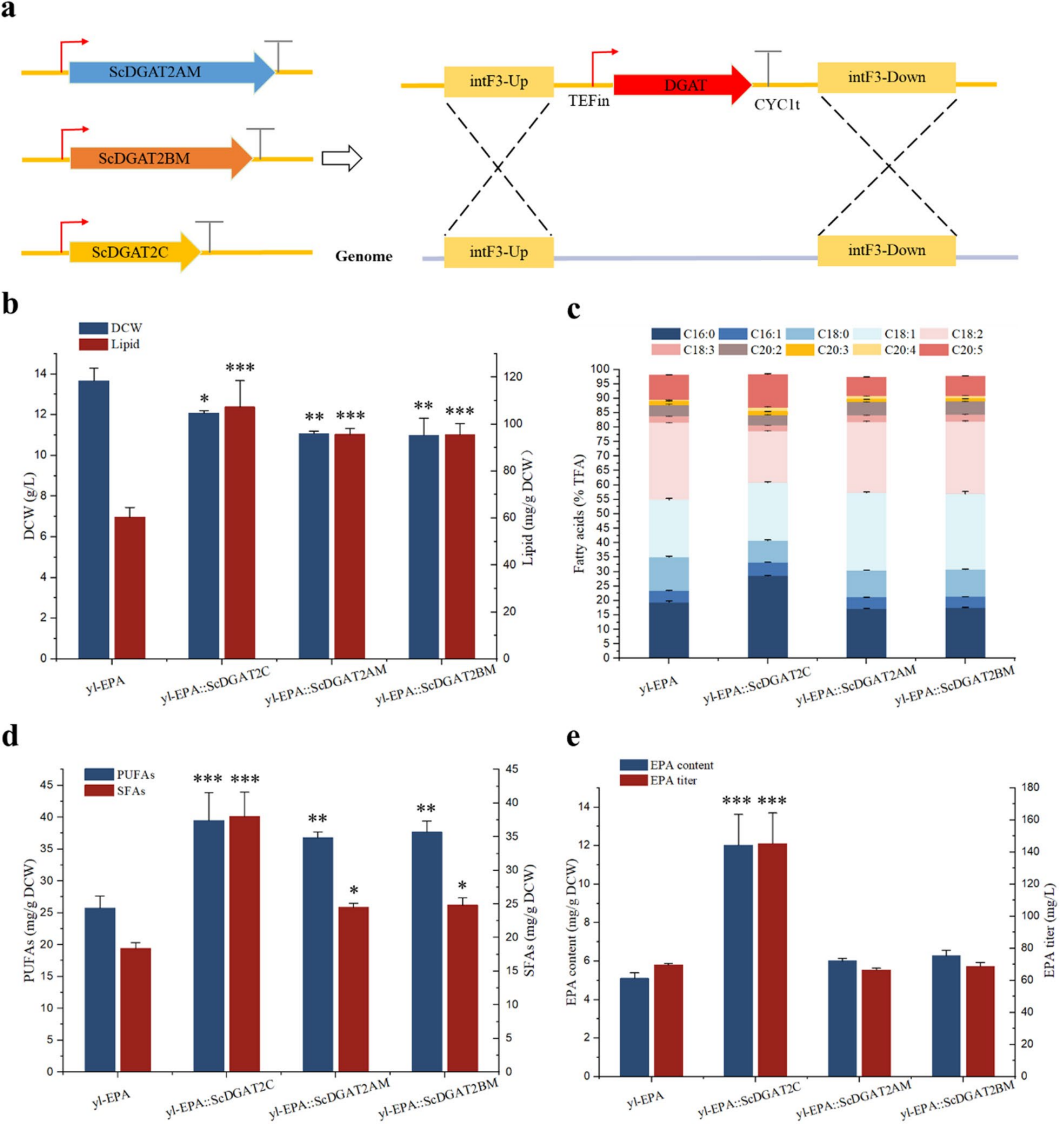
Effects of *DGAT*s on lipid and fatty acid synthesis in EPA-producing *Y. lipolytica* (named yl-EPA) strain. **a** Scheme of the expression of *DGAT* genes controlled by the TEFin promoter and CYC1t terminator at the intF3 integration site based on homologous recombination. intF3-Up, upstream homologous arm of integration site intF3; intF3-Down, downstream homologous arm of integration site intF3. Effects of *DGAT*s on lipid and DCW (**b**), fatty acid composition (**c**), UFAs and SFAs content (**d**), and EPA content and titer (**e**) in yl-EPA strain. Three biological replicates were used and mean values ± SD (n = 3) are shown. Two-way ANOVA with Tukey’s multiple comparisons test. *P < 0.05, **P < 0.01, and ***p < 0.001 compared with the yl-EPA

We explored the "push–pull-promote" strategy to enhance EPA synthesis. First, more EPA was pulled into the TG for storage by expression of *DGAT*s (pull strategy). According to reports, *DGAT2* plays an important role in introducing unusual fatty acids such as PUFAs into lipids and TG [29]. Due to the desaturase/elongase pathway in *Y. lipolytica* is incomplete, naturally producing mainly C16:0, C16:1, C18:0, C18:1, and C18:2. Therefore, to further evaluate the effect of *DGAT* on the accumulation of PUFAs, a *Y. lipolytica* mutant strain (named yl-EPA) capable of producing EPA was used, which had been obtained in our laboratory by transferring Δ12 desaturase, Δ9 elongase, Δ8 desaturase, Δ5 desaturase, and Δ17 desaturase into PO1f (Table 1). Subsequently, the *ScDGAT2C*, *ScDGAT2A*M, and *ScDGAT2BM* genes were integrated into the intF3 loci of the yl-EPA strain in the same manner as described above to investigate the effect of *DGAT* on PUFAs synthesis (Fig. 3a). As seen in Additional file 1: Fig. S6b, these *DGAT* genes had been successfully integrated into the genome of *Y. lipolytica* and the engineered strains yl-EPA::ScDGAT2C, yl-EPA::ScDGAT2AM, and yl-EPA::ScDGAT2BM were obtained.

Compared to the yl-EPA strain, yl-EPA::ScDGAT2C, yl-EPA::ScDGAT2AM, and yl-EPA::ScDGAT2BM strains showed a 13.16%, 23.38%, and 24.27% decrease in DCW and a 77.66%, 58.42%, and 58.29% increase in lipid content, respectively (Fig. 3b). In addition, the fatty acid profiles of yl-EPA::ScDGAT2C, yl-EPA::ScDGAT2AM, and yl-EPA::ScDGAT2BM strains were significantly changed compared to the yl-EPA strain (Fig. 3c, Additional file 1: Fig. S7). Compared to the yl-EPA strain, the C18:0 and C18:2 ratios in total fatty acids of ScDGAT2C strain were decreased by 48.27% and 50.31%, respectively. In contrast, the C16:0, C16:1, C20:4, and C20:5 ratios were increased by 47.35%, 15.17%, 173.08%, and 32.29%; Compared to the yl-EPA strain, the C16:0, C18:0, C20:3, and C20:5 ratios in total fatty acids of ScDGAT2AM strain were decreased by 12.52%, 23.98%, 36.96%, and 31.85%, respectively. In contrast, the C18:1, C20:2, and C20:4 ratios were increased by 35.32%, 18.62%, and 103.85%; compared to the yl-EPA strain, the C16:0, C18:0, C20:3, and C20:5 ratios in total fatty acids of ScDGAT2BM strain were decreased by 10.91%, 23.45%, 32.63%, and 26.62%, respectively. In contrast, the C18:1, C20:2, and C20:4 ratios were increased by 32.19%, 17.60%, and 103.85%.

Compared to the yl-EPA strain, the PUFAs and SFAs contents of yl-EPA::ScDGAT2C, yl-EPA::ScDGAT2AM, and yl-EPA::ScDGAT2BM strains were significantly increased (Fig. 3d). Compared to the yl-EPA strain, the PUFAs content of yl-EPA::ScDGAT2C, yl-EPA::ScDGAT2AM, and yl-EPA::ScDGAT2BM strains were increased by 53.52%, 43.10%, and 46.48%, respectively, and the SFAs content were increased by 106.63%, 33.04%, and 34.95%, respectively, indicating that the expression of *ScDGAT2C*, *ScDGAT2A*M, and *ScDGAT2B*M genes enhanced the assembly of PUFAs-TG and SFAs-TG in *Y. lipolytica*. Consistent with previous reports, the expression of *DGAT2* from *Brassica napus* in *C. reinhardtii* increased the PUFAs content to 12% [10].

In addition, the EPA content and titer of yl-EPA::ScDGAT2AM and yl-EPA::ScDGAT2BM strains were not significantly different compared to the yl-EPA strain, whereas the EPA content and titer of yl-EPA::ScDGAT2C strain were increased by 135.42% and 108.57%, respectively (Fig. 3e), which may be due to the weaker activity of *ScDGAT2AM* and *ScDGAT2BM* in transferring the acyl portion of EPA-CoA to DG for TG synthesis, resulting in the release of EPA from polar lipids that were not well incorporated into TG for storage and was instead transported to the β-oxidation pathway for degradation [8]. In contrast, the expression of *ScDGAT2C* provides a strong pulling power to mediate the incorporation of EPA into TG for storage and protection, resulting in the enrichment of EPA in TG and the increased level of TG-derived EPA. It has been reported that the expression of *CrDGTT1* from *Chromochloris zoftiensis* in *Nannochoropsis oceanica* increased the percentage of EPA in TG by 4.8-fold compared to the control strain. In addition, the EPA content derived from TG reached 10.3 mg/g DCW, which was 10.1-fold higher than of the control strain [8]. In addition to EPA, we also found that the expression of *ScDGAT2C*, *ScDGAT2AM*, and *ScDGAT2BM* genes resulted in an increase in C18:2, C18:3, C20:2, and C20:4 titer (Additional file 1: Fig. S8), suggesting that *ScDGAT2C*, *ScDGAT2AM*, and *ScDGAT2BM* may play an important role in incorporating these PUFAs into TG storage. Therefore, the application of these *DGAT* genes for metabolic engineering modification of other PUFAs synthesis could be considered in the future.

### Lipomics analysis of the effect of *ScDGAT2C* on EPA storage

**Fig. 4:**
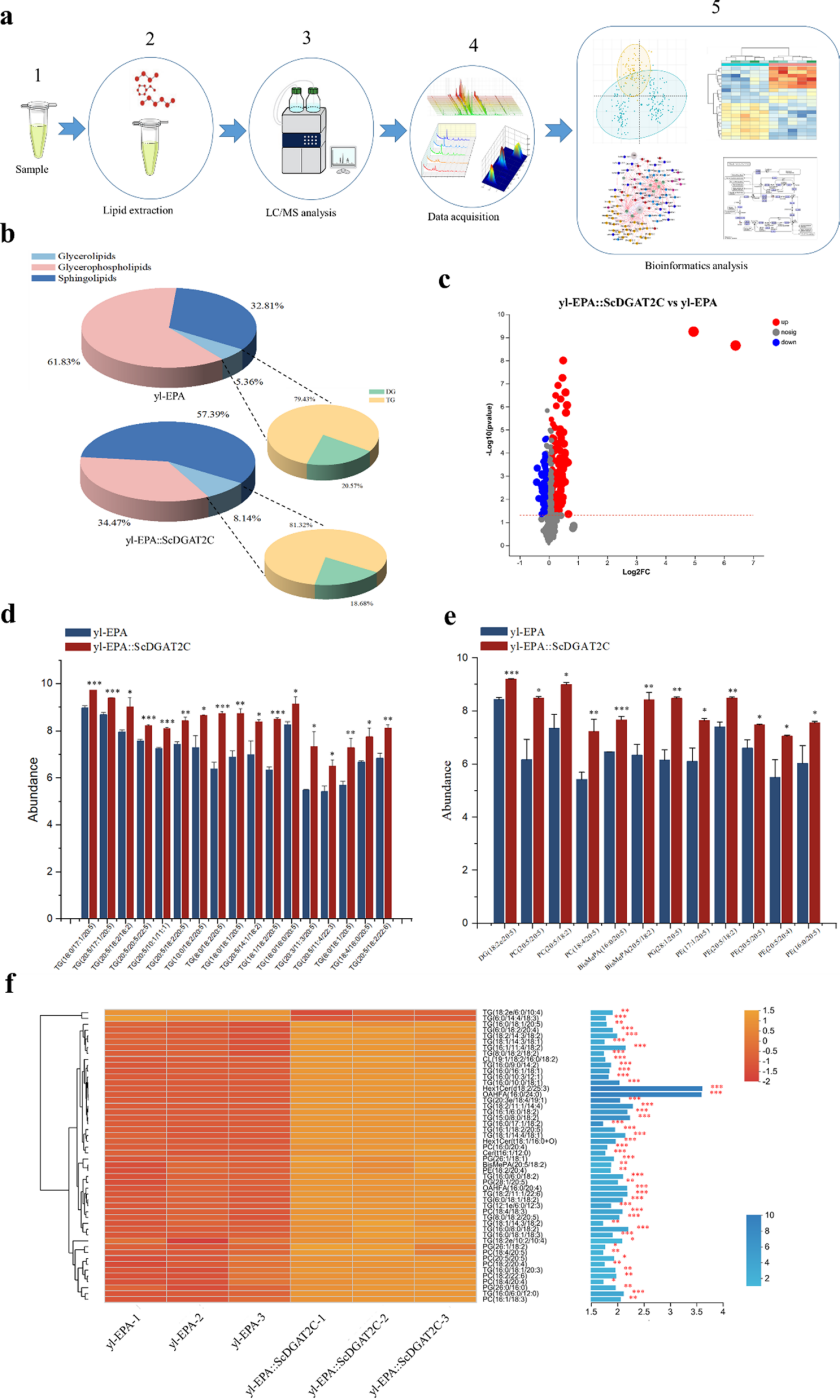
Lipomics analysis of the effect of Sc*DGAT*2C on EPA storage. **a** Schematic of the steps in the workflow of lipomics analysis. **b** Effects of Sc*DGAT*2C on glycerolipids, glycerophospholipids, sphingolipids, diacylglycerol (DG), and Triglyceride (TG) in yl-EPA strain. **c** Volcano plot analysis of yl-EPA::ScDGAT2C and yl-EPA. Red represents up-regulated metabolites, blue represents down-regulated metabolites, and gray represents unchanged metabolites. **d** Effects of Sc*DGAT*2C on EPA-related TG in yl-EPA strain. **e** Effects of Sc*DGAT*2C on EPA-related DG, phosphatidylcholine (PC), Bis-methyl phosphatidic acid (BisMePA), phosphatidylglycerol (PG), and phosphatidylethanolamine (PE) in yl-EPA strain. (**f**) Clustering analysis of lipid metabolites with the top 50 variable importance (VIP) values under Sc*DGAT*2C expression conditions. Each row of the color block gradient map on the left represents a metabolite, and the different colors represent the relative abundance of metabolites in the sample set. The right side is the VIP bar graph of metabolites, the length of the bar represents the contribution of the metabolite to the difference between two groups and the color of the bar indicates the significance of the difference. Three biological replicates were used and mean values ± SD (n = 3) are shown. Two-way ANOVA with Tukey’s multiple comparisons test. *P < 0.05, **P < 0.01, and ***p < 0.001 compared with the yl-EPA

Triglyceride/fatty acid (TG/FA) cycling is the process of partial or complete degradation of stored fat to release free FAs that subsequently are used to resynthesize a new molecule of TG [30]. According to reports, some marine microalgae such as *N. oceanica* and *P. tricornutom* can synthesize EPA, but it is mainly stored in polar membrane lipids rather than in lipid TG, which may be the reason for its limited content at a low level [8]. Unlike membrane lipids, TG is stored in lipid droplets and has the potential for high-level accumulation in microorganisms. In addition, the physiological properties and function of TG molecules depend on the composition of fatty acids attached to their glycerol backbone, which further highlights the potential application prospects of TG molecules. Therefore, channeling EPA to TG may be a promising way to overcome EPA storage limitations. Therefore, in order to further investigate the effect of *ScDGAT2C* on EPA storage in TG, lipidomics analysis was performed on yl-EPA and yl-EPA::ScDGAT2C strain. The experimental procedures included lipid extraction, LC/MS analysis, data acquisition, and bioinformatics analysis (Fig. 4a).

Glycerolipids, glycerophospholipids, and sphingolipids are the main components of lipid metabolites. As seen in Fig. 4b, the expression of *ScDGAT2C* resulted in a decrease in the relative content of glycerophospholipids from 61.83% of yl-EPA to 34.47%. In contrast, the relative contents of sphingolipids and glycerolipids increased from 32.81% and 5.36% of yl-EPA to 57.39% and 8.14%, respectively. In addition, the expression of *ScDGAT2C* resulted in an increase in the relative content of TG from 79.43% of yl-EPA to 81.32%, indicating that the expression of *ScDGAT2C* promoted the biosynthesis of TG.

Univariate statistical analysis (t-test) combined with multivariate statistical analysis (OPLS-DA/PLS-DA) and fold change (FC) were used to screen differential metabolites. There were a total of 189 differential metabolites between yl-EPA::ScDGAT2C and yl-EPA, of which 137 were upregulated and 52 were downregulated compared to the yl-EPA (Fig. 4c). TG is the major storage form of fatty acids. Although *DGAT*s plays an important role in promoting TG synthesis, there are some functional differences among different sources and types of *DGAT*s. For example, the functions of four *DGAT2*s (*DGAT2A*, *DGAT2B*, *DGAT2C*, and *DGAT2D*) in *Aurantiochytrium* sp. SD116 were investigated, and it was found that *DGAT2C* was mainly responsible for connecting PUFAs to the sn-3 position of the TG molecules, while *DGAT2A* and *DGAT2D* mediated the SFA-TGs production [5]. Comparative analysis of the differential metabolites between the two groups showed that 17 kinds of EPA-related TG were significantly increased compared to the yl-EPA strain, including TG (16:0/17:1/20:5), TG (20:5/17:1/20:5), TG (20:5/18:2/18:2), TG (20:5/20:5/22:5), TG (20:5/10:1/11:1), TG (20:5/18:2/20:5), TG (10:0/18:2/20:5), TG (8:0/18:2/20:5), TG (16:0/18:1/20:5), TG (20:5/14:1/18:2), TG (16:1/18:2/20:5), TG (16:0/16:0/20:5), TG (20:3/11:3/20:5), TG (20:5/11:4/22:3), TG (6:0/18:1/20:5), TG (18:4/16:0/20:5), and TG (20:5/18:2/22:6) (Fig. 4d), indicating that the expression of *ScDGAT2C* increased the storage of EPA in TG, which is similar to another study in which *CrDGTT1* from *Chlamydomonas reinhardtii* was expressed in *Nannochloropsis oceanica*, the percentage of EPA in TG and the content of TG derived EPA increased by 4.8-fold and 10.1-fold, respectively, compared to the control strain [8]. Although *ScDGAT2C* has provided a strong pull to incorporate more EPA into TG for storage, due to the natural fatty acid profile of *Y. lipolytica*, the C18:2 in PUFAs and the C16:0 in SFAs account for a high proportion of total fatty acids (Additional file 1: Fig. S9a, b). Therefore, desaturase with strong catalytic activity for C18:2 and C16:0 can be explored to promote EPA synthesis in the future. In addition, other storage forms associated with EPA, including DG (18:2e/20:5), PC (20:5/20:5), PC (20:5/18:2), PC (18:4/20:5), BisMePA (16:0/20:5), BisMePA (20:5/18:2), PG (28:1/20:5), PE (17:1/20:5), PE (20:5/18:2), PE (20:5/20:5), PE (20:5/20:4), and PE (16:0/20:5) were also significantly increased compared to the yl-EPA strain (Fig. 4e). In addition, we found that the TG associated with C18:2, 18:3, and 20:4 in yl-EPA::ScDGAT2C were also significantly increased compared to the yl-EPA strain (Additional file 1: Fig. S10).

Subsequently, cluster analysis was conducted on the lipid metabolites with the top 50 variable importance (VIP) values, and it was found that TG accounted for a total of 30 metabolites with significant contributions to the differences between yl-EPA and yl-EPA::ScDGAT2C (Fig. 4f), among which three kinds of TG were related to EPA, including TG (16:0/18:1/20:5), TG (16:1/18:2/20:5), and TG (8:0/18:2/20:5). In addition, other storage forms related to EPA, including BisMePA (20:5/18:2), PG (28:1/20:5), PC (18:4/20:5), and PC (20:5/20:5), were also lipid metabolites that contributed significantly to the difference.

### *GPAT*s further enhanced the storage of EPA in TG

**Fig. 5:**
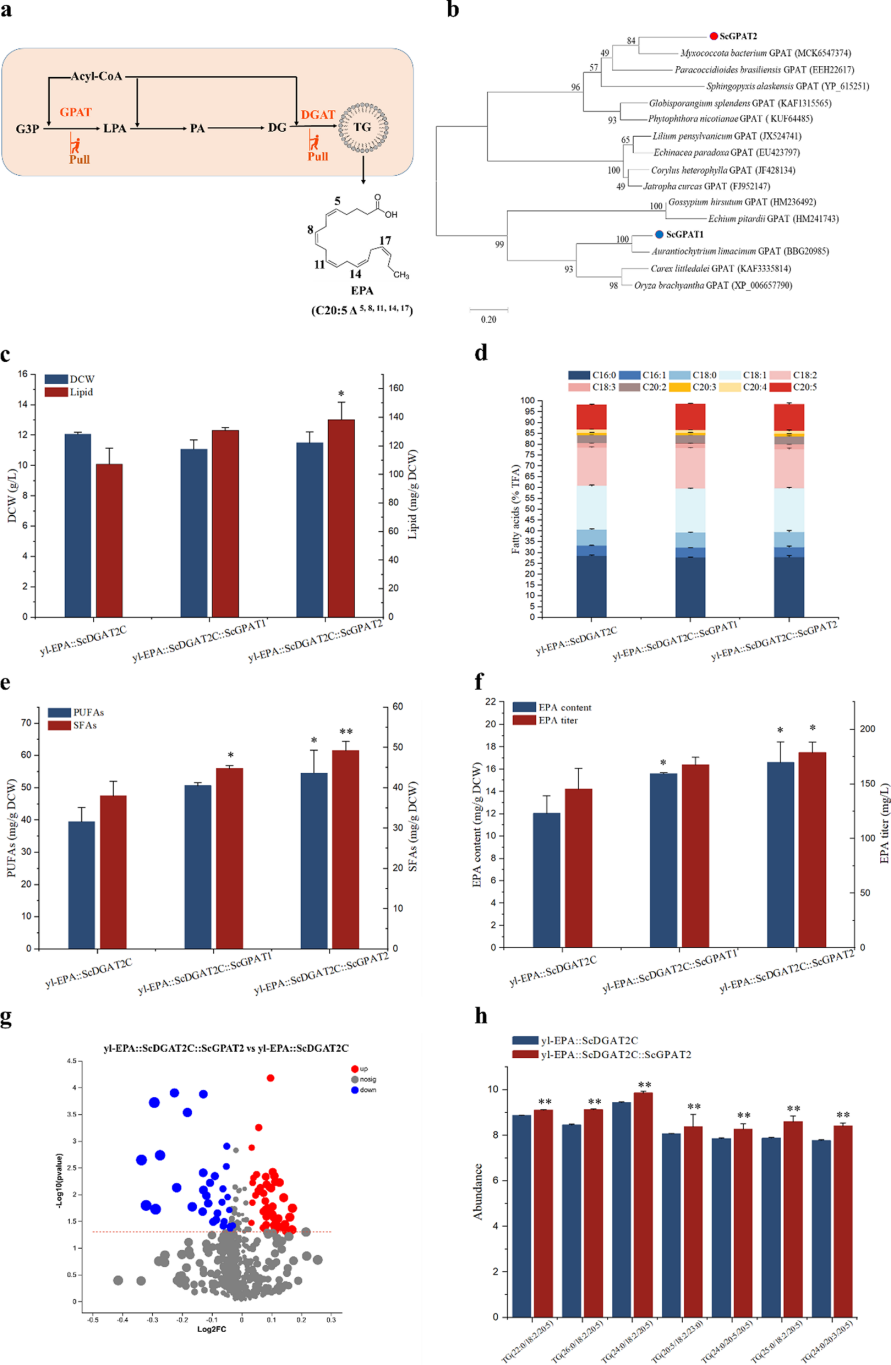
Effect of *GPAT*s on lipid and EPA synthesis. **a** Schematic diagram of the introduction of *GPAT*s to improve EPA storage. *EPA* eicosapentaenoic acid, *G3P* glycerol-3-phosphate, *GPAT* glycerol-sn-3-phosphate acyl-transferase, *LPA* lysophosphatidate, *PA* phosphatidate, *DG* diacylglycerol, *DGAT* diacylglycerol acyltransferase, *TG* triacylglycerol. **b** Phylogenetic analysis of amino acid sequences of *ScGPAT1* and *ScGPAT2*. Effects of *GPAT*s on lipid and DCW (**c**), fatty acid composition (**d**), PUFAs and SFAs content (**e**), and EPA content and titer (**f**) in yl-EPA::ScDGAT2C strain. **g** Volcano plot analysis of yl-EPA::ScDGAT2C::ScGPAT2 and yl-EPA::ScDGAT2C strains. Red represents up-regulated metabolites, blue represents down-regulated metabolites, and gray represents unchanged metabolites. **h** Effects of *ScGPAT2* on EPA-related TG in yl-EPA::ScDGAT2C strain. Three biological replicates were used and mean values ± SD (n = 3) are shown. Two-way ANOVA with Tukey’s multiple comparisons test. *P < 0.05, **P < 0.01, and ***p < 0.001 compared with the yl-EPA::ScDGAT2C

Due to its low activity and position as a first step, glycerol-3-phosphate acyltransferase (*GPAT*) is considered a rate-limiting enzyme in TG biosynthesis. Strategies to increase lipid accumulation by enhancing *GPAT* expression have been successfully applied in a variety of microalgal species [14]. According to reports, blocking *GPAT* expression leads to a decrease in lipid accumulation, suggesting its crucial role in TG formation [31]. The expression of *GPAT* gene in *Phaeodactylum triornuum* not only promoted oil body formation, but also stimulated a double increase in neutral lipid content [32]. Therefore, on the basis of the yl-EPA::ScDGAT2C strain, *GPATs* was further introduced to pull more EPA into the TG for storage (pull strategy). (Fig. 5a).

Based on functional annotation of the genome of *Schizochytrium* sp. HX-308 and the phylogenetic analysis of amino acid sequences, two *GPAT* genes, *ScGPAT1* (gene ID: A1558) and *ScGPAT2* (gene ID: A5476), were identified (Fig. 5b). Multiple-sequence alignment of *Schizochytrium* sp. HX-308 *GPAT*s was performed with other *GPAT* amino acid sequences. *ScGPAT1* and *ScGPAT2* were found to contain four highly conserved motifs that have been suggested to play a role in the activities of acyltransferases (Additional file 1: Fig. S11) [33]. In this work, compared to the yl-EPA::ScDGAT2C strain, the expression of *ScGPAT1* and *ScGPAT2* genes increased lipid content by 21.92% and 28.92%, respectively (Fig. 5c). According to reports, heterologous overexpression of *GPAT* from *Lophosphaera incise* in *C. reinhardtii* increased TG content to 50% of DCW without growth restriction [34]. Compared to the yl-EPA::ScDGAT2C strain, the fatty acid profiles in yl-EPA::ScDGAT2C::ScGPAT1 and yl-EPA::ScDGAT2C::ScGPAT2 strains did not show significant changes (Fig. 5d). However, the expression of *ScGPAT1* and *ScGPAT2* resulted in an increase of 28.38% and 38.30% in PUFAs content, and an increase of 17.83% and 29.59% in SFAs content, respectively (Fig. 5e). In addition, compared to the yl-EPA::ScDGAT2C strain, the expression of *ScGPAT1* resulted in a 29.68% and 15.32% increase in EPA content and titer, respectively, and the expression of *ScGPAT2* resulted in a 37.91% and 23.00% increase in EPA content and titer, respectively (Fig. 5f).

Subsequently, lipidomics analysis was performed on yl-EPA::ScDGAT2C and yl-EPA::ScDGAT2C::ScGPAT2 strain. There were a total of 77 differential metabolites between yl-EPA::ScDGAT2C and yl-EPA::ScDGAT2C::ScGPAT2 strain, of which 45 were upregulated and 32 were downregulated compared to the yl-EPA:: ScDGAT2C (Fig. 5g). Furthermore, seven EPA-related TG were found to significantly increased compared to the yl-EPA::ScDGAT2C strain, including TG (22:0/18:2/20:5), TG (26:0/18:2/20:5), TG (24:0/18:2/20:5), TG (20:5/18:2/23:0), TG(24:0/20:5/20:5), TG (25:0/18:2/20:5), and TG (24:0/20:3/20:5) (Fig. 5h).

### Enhancing the precursor and cofactor supply to improve EPA accumulation

**Fig. 6:**
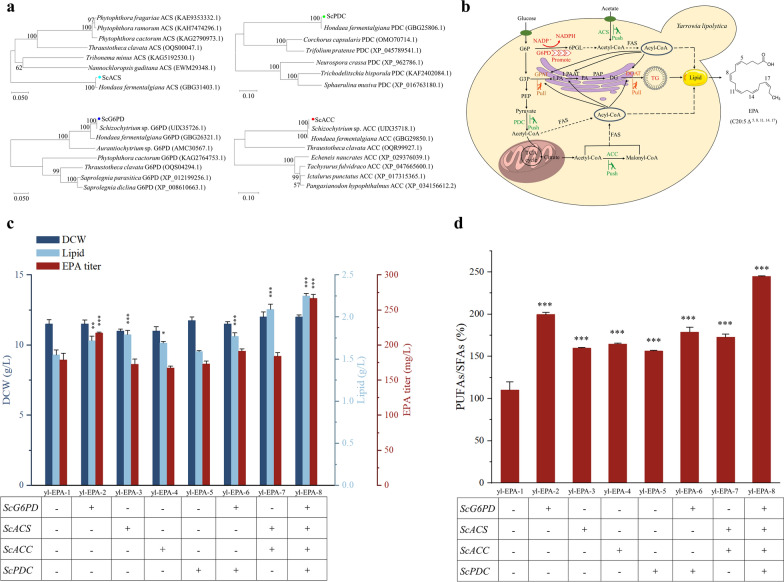
Effect of enhanced supply of precursors and cofactors on lipid and EPA accumulation. **a** Phylogenetic analysis of amino acid sequences of *ScACS*, *ScPDC*, *ScACC*, and *ScG6PD*. **b** Schematic diagram of metabolic engineering workflow for improved eicosapentaenoic acid synthesis in *Y. lipolytica* through a “push–pull-promote” strategy. *FAS* fatty acid synthase, *EPA* eicosapentaenoic acid, *ACS* acetyl-CoA synthetase, *PDC* pyruvate dehydrogenase complex, *ACC* acetyl-CoA carboxylase, *G6PD* glucose 6-phosphate dehydrogenase, *G6P* glucose-6-phosphate, *G3P* glycerol-3-phosphate, *GPAT* glycerol-3-phosphate acyltransferase, *LPA* lysophosphatidate, *LPAAT* lysophosphatidate acyltransferase, *PA* phosphatidate, *PAP* phosphatidic acid phosphatase, *DG* diacylglycerol, *DGAT* diacylglycerol acyltransferase, *TG* triacylglycerol, *PEP* phosphoenolpyruvate, *6PGL* 6-phosphogluconolactonase. Effects of *ScACS*, *ScPDC*, *ScACC*, and *ScG6PD* on lipid, DCW, EPA titer (**c**), and PUFAs to SFAs content ratios (**d**) in yl-EPA-1 strain. Three biological replicates were used and mean values ± SD (n = 3) are shown. Two-way ANOVA with Tukey’s multiple comparisons test. *P < 0.05, **P < 0.01, and ***p < 0.001 compared with the yl-EPA-1

Notably, in this study, although *DGATs* and *GPATs* provide pull to incorporate more EPA into TG storage, EPA was still below satisfactory levels due to the limitations of their lipid content [35]. Therefore, we further increase precursor and cofactor supply to enhance the synthesis of EPA and lipids. Acetyl-CoA is the actual form in which glycolytic pyruvate enters the tricarboxylic acid (TCA) cycle, as well as being an important precursor for fatty acid synthesis [36]. Acetyl-CoA synthetase (*ACS*) is an enzyme involved in carbon metabolism, which converts acetic acid into acetyl-CoA in an irreversible reaction [37]. It was reported that when the ACS gene from *Thalassiosira pseudonana* was heterologously expressed in the *Saccharomyces cerevisiae* fatty acid activation deletion strain (faa4D), the EPA level of the engineered strain was increased by more than 75-fold compared to the control [38]. Pyruvate dehydrogenase complex (*PDC*) connects the carbon flux from glycolysis with the tricarboxylic acid cycle by converting pyruvate into acetyl-CoA [39]. Acetyl-CoA from the cytoplasm is converted into malonyl-CoA by acetyl-CoA carboxylase (*ACC*). Subsequently, acetyl-CoA is used as the starting compound and malonyl-CoA as the elongation unit to continuously elongate fatty acids by C2 units until C16:0 or C18:0 acyl-CoA is synthesized via a type I fatty acid synthase (FAS). Besides, in addition to the initial two-carbon skeletons, fatty acid synthesis requires two NADPH molecules to extend the saturated C2 unit, as well as each double bond generated on the existing carbon skeleton [40]. Therefore, enhancing the supply of NADPH is not only beneficial for improving lipid synthesis but also for the synthesis of very long-chain PUFAs [41]. Glucose 6-phosphate dehydrogenase (*G6PD*) is the rate-limiting enzyme in the pentose phosphate pathway (PPP) that provides reducing power in the form of NADPH [22]. Based on functional annotation of the genome of *Schizochytrium* sp. HX-308 and the phylogenetic analysis of amino acid sequences, *ScACC* (gene ID: A2520), *ScACS* (gene ID: A2978), *ScPDC* (gene ID: A8969), and *ScG6PD* (gene ID: A6492) were identified (Fig. 6a). Therefore, based on the yl-EPA::ScDGAT2C::ScGPAT2 (named yl-EPA-1) strain, the supply of precursors required for lipid and EPA synthesis was enhanced by expression of *ScACC*, *ScACS*, and *ScPDC* (push strategy), as well as the supply of NADPH required for lipid and EPA synthesis was enhanced by expression of Sc*G6PD* (promote strategy) (Fig. 6b).

In this study, there was no significant difference in DCW of yl-EPA-2, yl-EPA-3, yl-EPA-4, yl-EPA-5, yl-EPA-6, yl-EPA-7, and yl-EPA-8 strains compared to yl-EPA-1 strains (Fig. 6c). However, the lipid titer of all strains except strain yl-EPA-5 was significantly increased compared to the yl-EPA-1 strain. The lipid titer of yl-EPA-2, yl-EPA-3, yl-EPA-4, yl-EPA-6, yl-EPA-7, and yl-EPA-8 strains were 1.72 ± 0.05 g/L, 1.79 ± 0.05 g/L, 1.69 ± 0.02 g/L, 1.77 ± 0.04 g/L, 2.09 ± 0.06 g/L, and 2.25 ± 0.03 g/L, respectively, which increased by 10.97%, 15.48%, 9.03%, 14.19%, 34.84%, and 45.16% compared to the yl-EPA-1 strain, respectively (Fig. 6c). Notably, the EPA titer of yl-EPA-2 and yl-EPA-8 strains reached 217.03 ± 1.22 and 266.44 ± 5.74 mg/L, respectively, an increase of 21.38% and 49.02% compared to the yl-EPA-1 strain, respectively (Fig. 6c). According to reports, the expression of *G6PD* from *Phaeodactylum tricornutum* in *Chlorella pyrenoidosa* resulted in an increase in NADPH content and a 24.78% increase in total PUFAs content compared to the wild type [42]. Consistent with previous reports, in this work, the expression of *ScG6PD* resulted in an increase in lipid and EPA titer (Fig. 6c). Moreover, the percentages of PUFAs such as C18:3, C20:2, C20:3, and C20:4 in total fatty acids of yl-EPA-8 strain were also increased significantly compared to the yl-EPA-1 strain (Additional file 1: Fig. S12). In addition, the PUFAs to SFAs content ratios of yl-EPA-2, yl-EPA-3, yl-EPA-4, yl-EPA-5, yl-EPA-6, yl-EPA-7, and yl-EPA-8 strains increased by 80.71%, 44.78%, 49.24%, 41.68%, 62.00%, 56.43%, and 121.78%, respectively, compared to the yl-EPA-1 strain (Fig. 6d). Therefore, the introduction of these genes further enhances the synthesis of lipids, PUFAs, and EPA.

## Conclusions

In this study, we explored the lipid synthesis genes of *Schizochytrium* sp. and constructed a *Y. lipolytica* cell factory through a rationally designed “push–pull-promote” metabolic engineering strategy for EPA production. The lipid and EPA titer of the final engineered strain reached 2.25 ± 0.03 g/L and 266.44 ± 5.74 mg/L, respectively, which increased by 174.39% (0.82 ± 0.02 g/L) and 282.27% (69.70 ± 0.80 mg/L) compared to the initial strain, respectively.

## Materials and methods

### Cloning and bioinformatics analysis of genes

The *ScDGAT2A*, *ScDGAT2B*, *ScDGAT2C*, *ScDGAT3*, *ScGPAT1*, *ScGPAT2*, *ScACS*, *ScACC*, *ScPDC* and *ScG6PD* genes were amplified by PCR from the genome of *Schizochytrium* HX-308. All the gene sequences were listed in Additional file 1: Table S1.

NCBI Conserved Domains Search (https:// www. ncbi. nlm. nih. Gov/ Struc ture/ cdd/ wrpsb. cgi) and TMHMM 2.0 (http:// www. cbs. dtu. dk/ services/ TMHMM/) were used to predict the conserved domains and transmembrane helices of *DGAT*s and *GPAT*s protein, respectively. Euk-mPLoc 2.0 was used to predict the subcellular localization of *DGAT*s protein (http://www.csbio.sjtu.edu.cn/bioinf/euk-multi-2/). The homology modeling of DGAT proteins was built by the Phyre2 web portal [43].

### Strains and media

All the strains and plasmids employed in this work are listed in Additional file 1: Table S2. *Schizochytrium* sp. HX-308 (CCTCC M209059) strain used in this work has been preserved in the China Center for Type Culture Collection (CCTCC) [44]. *Escherichia coli* DH5α was cultured in Luria–Bertani (LB) medium with ampicillin at 37 ℃ and 200 rpm. All *Y. lipolytica* transformants were grown in yeast peptone dextrose (YPD) medium at 30 ℃ and 220 rpm. The appropriate yeast transformants were isolated by using synthetic complete medium without uracil (SC-Ura) plates. The URA3 maker was recycled by selection on YPD plates supplemented with 1 g/L 5-fluoroorotic acid (5-FOA) and 15 g/L agar. The medium and conditions required for the fermentation of *Y. lipolytica* in this work were also described previously [45].

### Construction of recombinant plasmids and *Y. lipolytica* transformation

All the primers used in this work were designed based on the genome sequence of *Schizochytrium* HX-308. The *ScDGAT2A*, *ScDGAT2B*, *ScDGAT2C*, *ScDGAT3*, *ScGPAT1*, *ScGPAT2*, *ScACS*, *ScACC*, *ScPDC* and *ScG6PD* genes were cloned from the genome of *Schizochytrium* HX-308. Primer pairs ScDGAT2A-F/ScDGAT2A-R, ScDGAT2B-F/ScDGAT2B-R, ScDGAT2C-F/ScDGAT2C-R, ScDGAT3-F/ScDGAT3-R, ScGPAT1-F/ScGPAT1-R, ScGPAT2-F/ScGPAT2-R, ScACS-F/ScACS-R, ScACC-F/ScACC-R, ScPDC-F/ScPDC-R, and ScG6PD-F/ScG6PD-R were used to amplify the *ScDGAT2A*, *ScDGAT2B*, *ScDGAT2C*, *ScDGAT3*, *ScGPAT1*, *ScGPAT2*, *ScACS*, *ScACC*, *ScPDC* and *ScG6PD* genes, respectively. The resulting *ScDGAT2A*, *ScDGAT2B*, *ScDGAT2C*, and *ScDGAT3* gene fragments were then cloned into the pUC-intF3-HUH vector with the HisG-URA3-HisG blaster cassette using the ClonExpress MultiS One-Step Cloning Kit (Vazyme Biotech Co., Ltd., China), according to the manufacturer’s instruction, to generate the vector pUC-intF3-ScDGAT2A, pUC-intF3-ScDGAT2B, pUC-intF3-ScDGAT2C, and pUC-intF3-ScDGAT3, respectively. Similarly, the resulting *ScGPAT1*, *ScGPAT2*, *ScACS*, *ScACC*, *ScPDC* and *ScG6PD* gene fragments were cloned into the pUC-HUH vector with the HisG-URA3-HisG blaster cassette to generate vector pUC-HUH-ScGPAT1, pUC-HUH-ScGPAT2, pUC-HUH-ScACS, pUC-HUH-ScACC, pUC-HUH-ScPDC, and pUC-HUH -ScG6PD, respectively.

Then, when the OD_600_ of the strain to be transformed cultured in YPD medium reached between 0.8 and 1.0, the *Y. lipolytica* cells were centrifuged for transformation. The recombinant plasmids were transferred into *Y. lipolytica* by using the Frozen-EZ Yeast Transformation II Kit (Zymo Research, Orange, CA) according to the manufacturer’s instructions. The appropriate yeast transformants were isolated by using SC-Ura plates and the DNA of these transformants was isolated using an E.Z.N.A.^®^ Yeast DNA Kit (Omega Bio-Tek Inc., USA) and verified by PCR. The reagents and methods used for plasmid construction and transformation in this work were also described previously [45]. The modified DNA fragments and plasmids were sequenced by Sangon Biotech Co., Ltd. (Shanghai, China). The recombinant strain was incubated on YPD + 5-FOA plates for 2–3 days to remove the URA3 counter-selection marker, as strains expressing URA3 did not grow in the presence of 5-FOA. All the primers used for the genetic modification in this work are listed in Additional file 1: Table S3.

### DCW, total lipid, and fatty acid analysis

The methods of DCW determination, lipid extraction, and fatty acid methyl esters (FAMEs) in this work were described previously [45]. After the fermentation, 40 mL of the fermentation broth was centrifuged and washed twice with water, and then lyophilized for 48 h for DCW quantification. About 2 mg of lyophilized cell were weighed and 500 μL 1 M sodium hydroxide in methanol with 100 μL of heptadecanoic acid (C17:0) was added and shaken at 1200 rpm for 2 h. Subsequently, 40 μL of 98% sulfuric acid was added, followed by 400 μL of hexane and shaken at 1200 rpm for 10 min to extract fatty acid methyl ester (FAME). After centrifugation at 8000 rpm for 2 min, the top hexane layer was taken for GC analysis. The prepared FAMEs samples were analyzed by a GC-2030 gas chromatography system (GC-2030, Shimadzu, Japan) equipped with a flame ion detector (FID) and a DB-23 GC column (60.0 m × 0.25 mm × 0.25 μm, Agilent, USA).

### Lipidomics analysis

At the end of fermentation, samples were collected and centrifuged at 4 °C and 5000 g for 5 min, during which time they were quickly rinsed 2–3 times with pre-cooled PBS solution. The samples were then stored at -80 °C for subsequent lipidomics analysis.

The 50 mg sample was weighed into a 2 mL centrifuge tube, to which a 6 mm diameter grinding bead, 200 μL of extraction solution (isopropanol: acetonitrile = 1:1), and 400 μL of MTBE were added and placed in a frozen tissue grinder at − 10 °C for 6 min, followed by ultrasonic extraction at 5 °C for 30 min, and then stand at − 20 °C for 30 min. Next, the samples were centrifuged at 4 °C and 13000*g* for 15 min, 350 μL of supernatant was taken into a centrifuge tube and blown dry with nitrogen. Then, the samples were re-dissolve by adding 100 μL of extraction solution, followed by vortexing for 30 s and then ultrasonic extraction at 5 °C for 5 min. Subsequently, it was centrifuged at 4 °C and 13000*g* for 10 min and the supernatant was removed for lipidomics analysis. Lipidomics analysis was performed by Shanghai Majorbio Bio-pharm Biotechnology Co., Ltd. (Shanghai, China).

### Statistical analysis

Three independent replicates were performed for all experiments, with results presented as mean ± standard deviation (SD). Student’s t test and Two-way ANOVA with Tukey’s multiple comparisons test was used for statistical analysis. P values of < 0.05 were considered statistically significant, and statistical significance is indicated as *P < 0.05, **P < 0.01, and ***p < 0.001.

### Supplementary Information

**Additional file 1: Table S1.** Genes used in this study. **Table S2.** Strains and plasmids used in this study. **Table S3.** Primers used in this study. **Figure S1. **Biosynthesis pathway of triglyceride in* Schizochytrium* sp. HX-308. *FAS* fatty acid synthase, *PKS* polyketide synthase, *EPA* eicosapentaenoic acid, *DPA* docosapentenoic acid, *DHA* docosahexaenoic acid, *G3P* glycerol-3-phosphate, *GPAT* glycerol-3-phosphate acyltransferase, *LPA* lysophosphatidate, *LPAAT* lysophosphatidate acyltransferase, *PA* phosphatidate, *PAP* phosphatidic acid phosphatase, *DG* diacylglycerol, *DGAT* diacylglycerol acyltransferase, *TG* triacylglycerol. **Figure S2. **Amino acid sequence analysis of four *DGAT*s. **a** Phylogenetic analysis of amino acid sequences of* ScDGAT2A*, *ScDGAT2B*, *ScDGAT2C*, and* ScDGAT3*. The neighbor-joining method was used to reconstruct the cladogram under the software MEGA 7. The scale bar 0.2 represents 20% divergence. The bracket after the species name represents the GenBank ID. **b** Protein sequence alignment of *ScDGAT2A*, *ScDGAT2B*, and *ScDGAT2C* with *DGAT2*s from five organisms. **Figure S3. **Predicated transmembrane domains for *DGAT*s by TMHMM. **Figure S4.** Predicated subcellular localization of proteins for *DGAT*s. **Figure S5.** Conserved domains detected in *DGAT*s by NCBI Conserved Domains Search. **Figure S6.** PCR validation of *DGAT* genes integration into CK strain (**a**) and yl-EPA strain (**b**) genome. **Figure S7. **Gas chromatography (GC) analysis of *ScDGAT2C*, *ScDGAT2AM*, and *ScDGAT2BM* expressed sample.** Figure S8. **Effect of *DGAT*s expression on fatty acids titer. Three biological replicates were used and mean values ± SD (n=3) are shown. Two-way ANOVA with Tukey’s multiple comparisons test. *P < 0.05, **P < 0.01, and ***p < 0.001 compared with the yl-EPA-1. **Figure S9. **Effect of *ScDGAT2C* expression on PUFAs (**a**) and SFAs (**b**) composition in yl-EPA strain.** Figure S10. **Effect of *ScDGAT2C* expression on fatty acid composition in TG. Effects of Sc*DGAT*2C on TG associated with C18:2 (**a**), 18:3 (**b**), and 20:4 (**c**) in yl-EPA strain. Three biological replicates were used and mean values ± SD (n = 3) are shown. Student’s t test was used for statistical analysis, and statistical significance is indicated as *P < 0.05, **P < 0.01, and ***p < 0.001. **Figure S11.** Protein sequence alignment of *ScGPAT1* and *ScGPAT2* with *GPAT*s from five organisms. **Figure S12. **Comparison of fatty acid composition between yl-EPA-1 and yl-EPA-8 strains. *TFA* total fatty acids. Three biological replicates were used and mean values ± SD (n = 3) are shown. Student’s t test was used for statistical analysis, and statistical significance is indicated as *P < 0.05, **P < 0.01, and ***p < 0.001.

## Data Availability

All data generated or analysed during this study are included in this published article and its supplementary information files.

## Abbreviations

DCW: Dry cell weight
FAS: Fatty acid synthase
EPA: Eicosapentaenoic acid
ACS: Acetyl-CoA synthetase
PDC: Pyruvate dehydrogenase complex
ACC: Acetyl-CoA carboxylase
G6PD: Glucose 6-phosphate dehydrogenase
G6P: Glucose-6-phosphate
G3P: Glycerol-3-phosphate
GPAT: Glycerol-3-phosphate acyltransferase
LPA: Lysophosphatidate
LPAAT: Lysophosphatidate acyltransferase
PA: Phosphatidate
PAP: Phosphatidic acid phosphatase
DG: Diacylglycerol
DGAT: Diacylglycerol acyltransferase
TG: Triacylglycerol
PEP: Phosphoenolpyruvate
6PGL: 6-Phosphogluconolactonase
PUFAs: Polyunsaturated fatty acids
UFAs: Unsaturated fatty acids
SFAs: Saturated fatty acids
