# Supplementary material for: Identification of lipid synthesis genes in Schizochytrium sp. and their application in improving eicosapentaenoic acid synthesis in Yarrowia lipolytica
Source: Biotechnol Biofuels Bioprod. 2024 Feb 24;17:32. doi: 10.1186/s13068-024-02471-y (PMC10894473; doi:10.1186/s13068-024-02471-y)
Supplement: Supplementary file 1 — Additional file 1: Table S1. Genes used in this study. Table S2. Strains and plasmids used in this study. Table S3. Primers used in this study. Figure S1. Biosynthesis pathway of triglyceride in Schizochytrium sp. HX-308. FAS fatty acid synthase, PKS polyketide synthase, EPA eicosapentaenoic acid, DPA docosapentenoic acid, DHA docosahexaenoic acid, G3P glycerol-3-phosphate, GPAT glycerol-3-phosphate acyltransferase, LPA lysophosphatidate, LPAAT lysophosphatidate acyltransferase, PA phosphatidate, PAP phosphatidic acid phosphatase, DG diacylglycerol, DGAT diacylglycerol acyltransferase, TG triacylglycerol. Figure S2. Amino acid sequence analysis of four DGATs. a Phylogenetic analysis of amino acid sequences of ScDGAT2A, ScDGAT2B, ScDGAT2C, and ScDGAT3. The neighbor-joining method was used to reconstruct the cladogram under the software MEGA 7. The scale bar 0.2 represents 20% divergence. The bracket after the species name represents the GenBank ID. b Protein sequence alignment of ScDGAT2A, ScDGAT2B, and ScDGAT2C with DGAT2s from five organisms. Figure S3. Predicated transmembrane domains for DGATs by TMHMM. Figure S4. Predicated subcellular localization of proteins for DGATs. Figure S5. Conserved domains detected in DGATs by NCBI Conserved Domains Search. Figure S6. PCR validation of DGAT genes integration into CK strain (a) and yl-EPA strain (b) genome. Figure S7. Gas chromatography (GC) analysis of ScDGAT2C, ScDGAT2AM, and ScDGAT2BM expressed sample. Figure S8. Effect of DGATs expression on fatty acids titer. Three biological replicates were used and mean values ± SD (n=3) are shown. Two-way ANOVA with Tukey’s multiple comparisons test. *P < 0.05, **P < 0.01, and ***p < 0.001 compared with the yl-EPA-1. Figure S9. Effect of ScDGAT2C expression on PUFAs (a) and SFAs (b) composition in yl-EPA strain. Figure S10. Effect of ScDGAT2C expression on fatty acid composition in TG. Effects of ScDGAT2C on TG associated with C18:2 (a), 18:3 (b), and 20:4 (c) in yl-EPA strain. [file 13068_2024_2471_MOESM1_ESM.docx]

**Identification of lipid synthesis genes in *Schizochytrium* sp. and their application in improving eicosapentaenoic acid synthesis in *Yarrowia lipolytica***

Yu-Lei Jia ^a^, Qing-Ming Zhang ^a^, Fei Du ^a^, Wen-Qian Yang ^a^, Zi-Xu Zhang ^a^, Ying-Shuang Xu ^a^, Wang Ma ^a^, Xiao-Man Sun ^a^*, He Huang ^a^

^a^ School of Food Science and Pharmaceutical Engineering, Nanjing Normal University, Nanjing 210000, China

**Correspondence:** [xiaomansun@njnu.edu.cn](mailto:xiaomansun@njnu.edu.cn)

**Additional file 1: Table S1.** Genes used in this study.

| Names | Accession numbers | Sequences (5’ > 3’) |
| --- | --- | --- |
| *ScDGAT2A* | A6553 | ATGCTAGCGCGCAAGGTGGGCAGCCTGCTACGGCACTGGCCGCGCTGGTCGCTGCTGCTGGTCCTGGGACGCCACTTCGACGTGGTCGAGGAGTTCTGCCGTTGCGAGTCCGAGTTCCCGCGCAAACGCGCGCGCAGCGCCGCGGCCAGCGAGGAGGATGTCTTTGAGTTCAGGCGCGCGGCGCGGGCCGGCATGCGCGAGATTCCGGGCGGACGAGTCGACGTTGTCCTCGTCCTTCTGTGTCTCTCCGTCGCTCTCCTGCTTGTGCGCCACTTTGCCACGCCCGTCATGGTCGACCGCTTTCTGGGCACCGGCATCGCCCTTAGCGCGCTCGGCATCGTTGTGGGATTCCTTTTCGGGGTCACGGGCATCCAGGAGGAAACTCGGCTGTCCCAAGTCGCGCAGCAACGCCAAAACGCTGACGCGCGCAAAGACCAACCTTCGTCGGCGCGCAGCGTGCTCCTCACTGCGCTACAAAATGCAGTCTTGCTCGGCTGCTTCATCGTGTCGACTGCGCCCTTCACGCTGCCGTGGTTGCTCGCCTACTTTTTCCCCTTTGAGTTGCTCTGCTTTGTAGTATTCTTCACCATCATTCTCGAAGTAGGGAGCCAAGTCTCGGCCCTCTGCCACGCCCCGCCTGGCGCCGATTACCACCAGGACCAAATTCTGGAAGGGGACGGGACCGCTCAAGAAGAGATGAGCATTGACAGCTCGTCGGTGAGCAAAGATACCTCTTCCCGTGAATTTGAAGACAGAATTGATCAGGCTGGAAAGGATGAGAAGCGCCGCAAGTACCTCAAAACCTACCTTTTGTCGGACAGCGGACGGGAGTGGTGCGGCGCTTTCATTGCGCTAGAGTGCGGCGTCATTGGCTTTGCTTCACTTCTCTCCTTTGCGTGGGAGCTTCTCCTCGTCTGGAATCTGAGCTCGTCGGCCACAGATGAGCTCGGCTCAACGCGCCACCATCACATTCTCCAGAGCAAGACCAAGCACTTTGCCGGACTTGACTGGCAAATGAGCTATGTAATTGTCATGTGTGGCGTGGGTTCCACGTCGGCCTTTTTGCAAGCCTTTTGGCTCACATATGCCGCAGGAGGGCGCTGGCTTCACTTTGCGAATGGCTGGGCATTCTTCCAGCCCTTGCGCGGCGGCTCGACTTTTGTCTTTCTCCAAGTCGTCGCATGGAGTCTCTATGGCGTCTGTCTCTCACTGTCCGTCGAGCGTCTTGCGCCCGTCATCCTCGGAGCTCTGTCCTCTGCCTTCGGCATGCAGCTGTCCTCCGACCTCATTGCTTTTGCATCAAGCGTACTGTGGGTACAAGGCTCCCTTGCGCGCTTTGTTGGGGCCACCAGACTGCTCCCGGGCGCAGTGGGTGCTCTCTATGTCTTTGCAGAGATTGTTATGGTTCTCTCGCTCAGTCAATTCAAGCGCTTCGAGCGTGATGAGGGACTTGTGCGCGAGGTAGTGACCACGAAGCACCGCGGGGCTCGCGTCCAGAGTCTTGTCAGAGAGCGCGAAGCGGAAGAAGGCCACTTTCTGTCCGGGTTGAGGGACATCATGCGAACAGCTTTCATGGCGTGCTTGGCCGTCTGTAGCGTAGAGCCCCACATTATCCTTTCGGGCGTCTCTGTGTGTTTCTTTCACTTGTTTGGCGATGGCGCTACGTCAATTTACGCGGTGTGCACGTGCGGCGCCATTTATCTCACAACCTATCTGGGAAATCCGAGCATCAGAGGCACACGGCGCTTGGATCCCGGATCATTCCTTTTTCGCATTGTTGAAGACCATTTCAGCCTCTCGATGGTGCGCACGTCTGAAGAGCCTTGGGACCCGGAGCAGGAGTACATTTGTGGCTATCACCCTCACGGCCTCGTGCCCTTGGGCGCCGCTTACATGAAAATGACCCCACAATGGTCGGAGCTCCTCCCCAATATTGTGCCCTTTACTCTCAGCGCAAGCATTACGCATCAAGTACCCATTTTGCGTGACTTTGTACAGCTAAGTGGAGGTCTCGAGGTGAGCAAAAAGGGAATCTGGGCAGGTCTTAACCGTTTTCGAAGCGTCGTGCTTGTGCCGGGAGGCCAGCACGAAATGCTCTTGGCGCCCTCGCGAGATCTCGAGGGCGTTGAGGAGCCCATTAGCACGAAACACAAGGGTTTCGTGCGCATCGCCTTTCGCCGTGCTGCGCGCCAACCCGACCGTAAGTTGTACTTGGTTCCTATTTTTGCTTTTGGTGACCGTCGCGCGCTACGGAATTTGCCCATCATCCCGAAGCGGGTCCAGCGCTGGTTCGTTCGCCGTATGCGAACGAATCCTGCAATGTTCCCCGTCGGCCGGTTCTCTCTCCCCTGCATTCCAGATCGCGGCCCCGTCACGATCGTTTTGGGGGATCCAATCGAGGTCCCCGTGCTCAAGGGCGGCGAGCGTGCTGTGCCAACAGAGGAGCAGGTGGATCTCTTCCACCGTAGGTATTACTCTCGACTCAAGGACATTTTTGACGAACACAAAGGCGTTCACGGCAATCAGTACGAGACGGCAAAGATGGTCTTTGTGCCGGAGCTGGAAACTGTTTCCGAGACAAGCTTTGAAGCGCAGTGGGCCAAGGTCGCTCACGATCACGACGATGAAGAGGAAGAAGATGCACTCGACTTTGAACGTGAAATCGACGAATCCATTTACGTTTGGCCTTGGCGAGAGCAGATTCTCGTCGTCTTCATTGTCATCTTCACCAGTTTTGGCCCAATTGCCGCCCTCTCCATTCTTTCTTCGGAAGCTACCCCTTATCCCAACATCACACTCCCCACCTTTTAG |
| *ScDGAT2B* | A7082 | ATGGAGGCCGCCAAGAACGAGGCTGCAGACTTTGCCAACCTGCGGCTCGAGCACGAGGCTTTGCAGAAACGATACGATGCGCTCGAGGCAGCGTACGCCAAGTTGCTCCTGGCCAAGGGCTCCTCCGAGGAGACCGACAAGACCGCGGAGAAGCCGTCGAGCTTTGCGGTCTGGAGCCCGTACATCAAAATCGCCGTTCTCTCGTCCGTGACTGCGCTGCTACTATTCATCTTTGCCGAGCGCCAGTACACCTCGGGTCAAAAGGTGGGCTTCGGCTTCCTCGCAGCCGTCGGCGGCGCGGGCGTGTTTTTAGCCATTCCCGCGACGCACTGGATCGGACACTTTTTGCACGATGACTACAAGATCTGGCAACCTTTTCGCGGCGGCGTGCGCTTCGTCGTGCTGCAGGCCATCTCATGGACATTTTACGGCATCACCGCGGTGATTGTCATGGCAGCCGTGGCTTTCGCCGAACATAATAATGGTATGCTCGCGAGCGCTGGCGTCGTCGGCCTTCTCTCGCAGGTCTTCATGGTGTCCTCGCTGCTAACATACAGCGACCCATCGCAGACGGCCCGCAAGGCACGCCGCATTTGGCGACAAAACAGCGGCGTCATCAATGCCGACGACCCATCCGATGAGGTCGAGGAAGAGGCGCGCCAGGCATACATGTCGCAACGCAGACCCTCGACGGACAGCGAGTATAGTTCCGGCTCTGGATCCACGTCCGCGACGGATAGGTCTGCACGTACGACGATGCGTCGTCGGTCTGTTGCCAAGTCGATCATCGACCATGACGCTGGGGCAAACGAGCAGCAGGTTCCCTTGGACAAAGACCACAGCACATCGAGCATCGTCAAGGAATTTGTGCAAATGAACACCTTTCTCGTTGTCATTGGCATTGTGCTAGGTGTTCTCTCCGAGCACAGCCCGGACAGCAAGCACACACCCATCTTTGGTATCCTTTCGCTCGTTTGCTGCATCGTGGCCATATCGCTTACACATGGCATCGGCGGTCGCTTGCGTCACATTAACCGCGGCTGGTCGTTTGCGCAGTTCTTCCGCGGCGGCAAAGAGTTCATTCTTTTGCAAATCTTTGGATGGTCCTTTTTCGGCGTTGCTGTCGTCGCACAAGGCGCCTTCGTGCTCTCGTCTGTTTACCTCGGCACGCACGGCATGAAAGGGACCATGTATGTGGGCGCGCTTGCTACTGTGATCTCGCACTATGCCATCGTGAGCTCGCTCCCTCGATTCGAGGACAGTGGCCCAATGCGTCCGACCATCACGCCTCGCAACTACCGTCCTCTGGAAGCCTTTACGCTTCGCGAACATTTCATGGTTTGGCCCATGATGATGCTCTTTTGCAACTTGCAGTTTCTCAGTTTTGTGCTCTACATGATTCTCTTTTGCGCGCCGTACGTCATGCCTGAGTCTTCGCAGCCATGGCTGGCCGAGGCCACCTACGGACTCCTTCCCGAGCTCTCTGCGAACTCGGCAGAACTTGGGCACACCTTCAAGGTTTGGTGGATTCTGCTCTGCGCCATCTCCTTTTGGACCTTTTTCTACTCGGCGCTCAATACCTGGGGAATCGATGGCTGGCGCCAAAGCCTCTTGCTTTCGCTCGCAGCTGTCGCAGCCTATGGTGGTGTGATTGCGGTTTTTCGCGAGTCTCCGCACTATGCCATGTTGGTCGTCATTTGCTCGGCGAACCTCGTCTACATTTCCACGACCTTTACCAAGCGTCCCGAGTACAATGCGTGTCGCGAGTGGTCAATTTTCAAGGAAATTGACGTGCTTCCTCGCCTCGCGGAAAAGTTCTTTGGATTGCGTCTCCAGCTCACCGAGGAAATGCAGCGTCTCGCGCCAGTTCTTGGCGATGTAAATGCCAAAGACCCCAAGAATCAGCAGGTGCTGCTTCTCTTTCACCCGCACGGCATCTTGCCCGTGACGCATGGCATCTTGCAGATTACACATGTGTGGCGCAAGATCTTTCCCCACCTCGACACAAATCCTCTCACTGCCACAATTACACATGTAGTGCCCGTGATGCGCGACGTTATTCAGTGGATGGGCTGCTGCGACGTTTCGCGAGCCACTGTGCATAATCTCATTCGAATGGGCCGCAACGTGCAGATCGTTTGCGGCGGCCAAACAGAAATGTTCGAGTCGCGTTCCTGGGACAACCGGATTGCCATCGTGCGAAAGCGTCGCCGCGGAATCTTCAAGATCGCCATTCAGCAAGGCCTCGGGATTGTGCCCATGTTCAGCTTTGGCGAGCCACAGATTTTCGATAATGTCTACATGCCGCGCACGCAGGCGTTTTTCAAGAATTTGCTGGGCTTTCCGTTCCCAATCTTCATGCTCGGAAAGTTTGGTCTGCCCATTCCCTGTCGCGTGCCCGTCACTGTCGCATTAGACGCGCCGGTGCATCCCGTTCGCCAGACAGCCAATCCAACGCCAGAGGAAATTTCCGAGTTCCAAGATCGTTACTTTGCCACGCTTGAGGCACTTTTCGAGCGCTACAAAGAAGAAAATGGCCATGGTTCTCATGAGCTTTCTTTCATTGACAATTGA |
| *ScDGAT2C* | A7100 | ATGCAGACACCGTACAGCACGTCGATGGGCTCCGTCTCGAGCTACTCGTCGTCTGGCGACTACGCCATGTCCGAAGCGGAGGGCTCCGAAGTGGACCCTGCCGATGACAGGATGGGCGACTCAATCGGATCGAAACCAAGCTCCTCGTCCGTGACCGGACGCCGGCGACTCACGCAAGAAGAGCGCGACTACTTTTTGCGTCTTGAAAAGGAATGGCGCGAGGAGGACGCATGGGCTGACCAACCCGGGTCCTGGTACTCAATGCTGGCCTGGATGCCGGTCCTCATTGGCCTGCGCGTCTTCAACGTGCTGCTTTCCATCGCCTTTTGGCCTGTCTCCTTTGTGGCGCGGGTCTTCTTCGGCAAAAAGATTCACACGGTCAGTTTCTGGGACGTTCCGCTTTCCCGCCGTAAACAAACTGCCGTGGTGCTCTTGTTCGTCATGCTCCTGCCCATGGTCGTCGTGGTGTACTCGTGGACGCTCGTTCTGCTTCTCTTCCCGCTCACGACCTTTCCCACACTGTGCTACATGGTGTGGATCATTTACGTGGACAAGAGTCCCGAAACAGGCTCGCGCCGACCCTTTTTGCGCTATTGGAAGATGTGGCGCCATTTCGCGAACTATTTCCCGCTTCGACTCATTCGCACCACGCCGCTCGACTCCCGTCGCAAGTACGTCTTTTGCTACCATCCGCACGGAATCATCTCGCTCGGTGCCTTTGGCAACTTTGCGACGGACTCGACCGGATTCTCGCGCAAATTCCCCGGTATCGATTTGCGCGTGCTCACGCTTGCGCTCAACTTTTACTGCCCCCTTTTGCGCGAGTTTCTGCTTTACCTGGGCCTTTGCAGCGCCGCAAAGAAGTCCTGCAACCAAATTTTGCAGCGCGGGCCTGGATCCGCCATCATGCTCGTCGTTGGCGGCGCCGCCGAGTCTCTCGACTCCCAGCCAGGCACCTATCGATTGACGCTCGGCCGAAAGGGGTTCGTGCGCGTTGCCCTTGACAATGGTGCCGATCTAGTGCCGGTGCTTGCCTTTGGCGAGAATGACGTATTTGACACCGTGTACCTCCCACCGAACTCGTGGGCACGAAACGTGCAAGAGTTTGTGCGCAAGAAGCTTGGTTTCGCCACACCAATTTTCAGTGGCCGCGGTATCTTCCAGTACAACATGGGGCTCATGCCTCACCGCCGTCCCATTATTGTCGTTGTTGGAAAACCCATCAAAATGCCCAAAATTCCCGATGAGCTCAAGGGCCGCGCGCTTTCCACCACGGCCGAGGGCGTTGCCCTCGTTGACAAGTACCACGAAAAGTATGTCAAGGCGCTTCGCGAGCTCTGGAACTTGTACAAGGAGCGCTGGGCCGTGCACCGTCAAGGTTCGCTGCTCATTCAAAAGTAA |
| *ScDGAT3* | A7950 | ATGACCCTGACCGGGCCGGAGGAAGACTACGCCGCGAGTCTGAGTCTGCAAGAGGACGCGGGCGCGCCCGAAGTCGCGGACGCCTCGGCGAGGGAGCGCAAGCAGCGCGCGCGAGATGCGCCAAGCTTGTTGCGCCAGGGCTCGTCCCAGGAAGACGCTGACAATACGCTCGTGCAGCAGACAGGAGTGGCGACTGTCCTCTTGCACGATGCCGAGGAGGTGGTGCGACAGGCGACCTTTGGGTCCGGACATCGCCACGCCGTGGCCATGGAGAATGTAGAAATTAAAACACCTGCGAAAACCCTCCCCGAAGGCACAACGCGCAAGCCCGTCCTCATTATCCCTGGCTTCATGTCGAGTTCGCTCCGTGTCGAGTCATCGAGTGTAGTGCCTCGATGGGAGGGTAAGCGCATTTGGATGTCGCTCGGGCGTTTGGGTTTCACGGGCAAATTTTTAGGAACGTCGAGCGTTTTTGAGACCAACGACGACGATGCGGAGCAGATCAGTATGCGGAATGATTGGCTCTTGCACATGTCACTTCAAACGGACCTCATTTCTGAGCGAGAAGGTGTGCGGGTGCGCGCCATTCCGGGCTTGCGTGGTGTTGATTTTCTCGAACCAGGTCTCTTCATGAACGCCCAGACCTACGTATTCGGGCCCGTAATTAGCGCTCTCGTCAAGCGCGGTGGTTACACTCCAGAAAAGGATCTCGATGCCGCATCGTACGACTGGCGCATGCCGCCTCGCATTCTCGAGGAGAGAGACCAATACTTTACGCGTACACTTGATCGCATCGAGCGTATGTGCCAAGAAAACGACAATCGTCGCGTCGTTCTTCTCTGTCACTCCATGGGCTGCCAAATGGGCGAATATCTTTTGCGTTTTGCACTGGATCGTCGCGGACGCGAGTGGATCGATCAGCACATCGAAACGTATCTTCCCGTGGGCGGTCCGCATCTCGGATCTCCGAGCGCCTTGCAGAGCCTCGTGCATGGATCTAACATGGGTCTTCCCGCGGCCTTTCTCTCTTCGCATGCTGCTCTCATCATGGGCCGGTCCTTGGGGTCGACTCCTTTCCTGTTGCCAGTGGCCACATCTGGAGATATCGAAGACGACCATACCGCAGCGAATTGCTTGTATCCTAACATCGTCAAGCAGACGGGCATGGTTCGCTTCAAAATCACCAAAATCGATCTTCGCCAAATCGCATCCTTTTATCGCAACTTGGGCCAGCTCCGTCTGCGCATTCGCTTCGGGCCCACGACCCTTGCAACCGCATGGTATACGGCGCACCCCATTCATCCGATTCGTCCCGTGGACGGCGACAACAATTACGTCATGTTCGAAATGGAGGCCCCTGTCGAGCTAGGCCAGGGGGATGACATCTTCATTGTCGAGATCGTCGAGCAGGTTCTTGCACTAGACGTTACAGCGCGTCGCCTATACCTTCCCAATCGCATCACGCGCTGCATGTGCGTCGACACCAAGCTCGGCAAGGCCAGCAGTGCTGCCTTTGAGAATTCTCTCGGCACTCTCAAAGTCATTGACCCTGGAACGGTTCTATGCAAGAACAAATTTCAGCTCGCGCACATTTTGCGCAACCACGATTCTGAAAATCCGCATTCCGAGCCTGGCACGCCTAAACTCTTTACTTTTCCTCTCGCCAACATAAAAACCTCCCGCACGCGATATGGGTGCTATGCAGAGGCAGAAATGGAAATCACTTGGTTCTCGCCTGAAACGCTCTACCAAGAAGCCGGCGCGGAGATGCCTCGACACGCTGCGCCCCTATATAAAACCAAGCGTCGCAAAAATGCATTTAACGCGGCGAGCTCAAGACCTCTGCTCCGCCTGTCGAAAACGTATACGCCATCCATGCCGTCAATGTTGACACTGTGA |
| *ScGPAT1* | A1558 | ATGAGCGACCACGACAACAACGACGACATCCCCAAGATGAACGGCGCAGTGGACGCCGCTGTCGACCAAGACGAAAATGCCGACTCCACATTTGACGCCAACGCCGCCGAGACCGACAAGAATGGAGAAGCCGAGGCAGATGCAGATGCAAAAGCCAAAGCCGAAGTCGAAGATGAAGATGCTGTTGCGGAAGAAGATGAAAACGGAGACACCGTCTCGCCGCTCACGGCAGCCTACCTTTTCTCGCGCCCGGGGCTGCCCTCGGACCCGCGAAGCCTCCCTGCTGCCAGGAGCTCGGCCGGCAGCACCGCTTCATCCTCCGAAGGAGCTGAGGGAGACGAACGTCAACACGACTTTCGCGAAGCCCTCAGCCGCACAACCAGCGAAGAAGTTGGCTACGATGTCGATATCGGTGGCGATGGCGACCAAGACCAAAACGGGGACGATGGCCACTATGCGACCGGATACAATGTCGACCACGTCGACGATGAAAATGACGATGACGATGACAGCGGCGAAAATGAGCTCAAGTCACCTTTGAGCGGCGCCAACCACGCGTCCCTTAGCAAAGCGCGCAGAGACACGCAGTCCTCCTTCGCTTCGCTCATGAGCCTGGGCACCGACGAAGACGACAATGCTGGCTTTGACCCGCGTCGCGTCGGCATGGAGAACGCAGTCTTTGGCGACTACGACTTGAAAAAGAGTCAATCCACCCAAGATTTCCTCCTCTCGTACATTGACGACGAAATGGCGCGACGAAACTTTCCTGGTCGTCTCTCAGTCATTGCCAACGAGAGCCTGACCGCACGTCGTCGACGCCGTCGTGTTCACGAAACCGGTGCTGATTCCGTCCCCGAGGGCGACGAGGATACGAGCGACACCGAAGATGCCGCTGCTTCCCCGGGACGCGGGGCGGCGGGCCAATCTGGTCAGGAGTCCAAGACTGGCGCCGCCACGAGCCCGCCCACATCCCCTACTGCGGCCTCGTCTGCATCGCCCGTTTTCCACGACTCTGTCGCCTCTCCGGATGCGAAGACCAAAGTCATTGGCGGCGCCTCTGCAACCCCAGAACAGCCCGCCGAGTCTGCGCCGTCCACGCCGCGACACAGGAAACCTGAATCCAAGCTGGCTGCTGAAATCTTTGGCGCCGCTGCTGATGTCTACATGGAGGGCATCACCTCCATCGTTGACGACAACTTTTGGAAGTGTTTCGAAAGCAAGAAGCCGCGTCCTTGGAACTGGAACGCGTATCTTTGGCCCATGTGGGGCCTCGGCGTCCTGGTGCGCTACTTTGTGCTCTTCCCTCTGCGCATTGTAATCTTCGTGCTTGGCTGGGTGCTCTTTGGCATTGGTATGTTCGTCGTGCAAATGTTCTTCAGCAAAGGCGAACAGCGCACAAACCTCGAGCACCGCCTCATCATGATGATGTGCGGCATCTTCTGTATCACATGGGGCGCTGTCATCCGTTACCACGGCTCCTACGTCAAGCCAAAGCCCGGACAGTGCCATCCGGTCTACGTCGCGAACCATACCTCAATGATCGACGTCATCATTTTGCAGCAGATGCGCTGCTTTTCCCTTGTCGGTCAGCGCCACAAAGGTATTGTGCGCTTTTTGCAAGAAGTCGTTCTCAGCACGCTTCAGTGCGTGTGGTTTGACCGTGGCGAGATCAAAGATCGGGCTGTTGTTGCGCAAAAGCTCAAGGAACACGCCCTTGACCCGGACCAAAATCCTCTTCTCGTCTTCCCCGAGGGAACATGCGTCAACAACGAGTACATTATCCAGTTCAAGAAGGGCATCTTCGAGATTGGCGTCCCCGTCGTGCCCGTCGCCATCAAGTACAACAAGCTCTTTGTCGATCCATTTTGGAATTCCCGCCAGCAATCCTTTCCCATGCATCTCGTCGAGCTCATGACGTCATTTTGCCTCATCTGCGACGTATGGTATTTGAAACCGCACGTCCGTCGTCCGGGCGAATCCTCGACACAGTTTGCTGCTCGTGTGAAGAAGTCCATTGCCGACAAGTCCGGGCTCAAAAACGTCAACTGGGATGGCTACATGAAGTACTGGAAACCATCAGCACGCTACTTGCATGCCCGCCAGGAGATCATCGCCGACAAGCTTCGCCGCATTCATCTTCCAGATCGCGGATCCACGCCTGGTCTCCACAGCGGTTCGGAGGGTCCCGACGATGATCAAGACGATCACTCGCAGTCCAAAACGCGTCTGCGCAAGACAAGCTCCATGTAG |
| *ScGPAT2* | A5476 | ATGCTCCGGCAAGGGTCCGCACGCAAGACTCGAGTCGTCGACGTCATGCACGAGGCGTGGAAAACCCGCGCCTCGCGCGAAAACGCCTCCCCGACGCTCGAAGAATCCATCTCTTGGTGGCGCCGCATCGCCGCTCTCCTGACCAGCGACCTGTGGTTCGCACTCGGACGCTACAAGGGCGGAGGCCTCTTGGGCCCACCGCTCTCCATCCAGCAAATGTACAATGCCAAAGTAGAAATCGAACCGACACAAAGCCCCTCGGAAGGCTTCGAGAGCAGCTGCGGGCGCGCTGGTGACGAGGAAGAGCTCGACTCGAGTCTGCAGCTCATCAGCAAACGCATGAAAGAGATCCTCAGCCGCATCGAGGGCCGCTGCCGTATGTCTGCGGTCCGTTTTCTCGGCTGGATCCTCGGCAAGCTTTGGAAAGCCATCTTTTCCGCCATCGAAGTCGACCTTGAGGGTGTAAGTCGCGCGCGCGAAGGCCTCGAACGCGCAGCGCGCTCGGGAAGCGCAGTCATGCTCTTGCCAACGCATCGCAGCCACATTGATTACCTCCTCATGTCTTACTTGCACTTTGGCTTCAACTTTGCCGTGCCGCACATCGCATCGGGCGACAACCTCAACATTCCCGTCATCGGACCTCTTTTCTCATACTCTGGCGCCTTCTTTCTGCGACGATCGTTTCGCGGTGACAACCTCTACAAAAAGGTCCTTTACGGCTACCTGCTGCGCAAATTGCGCGAAGGCGCCCCCGTCGAGGTCTTCATCGAGGGCGGACGTAGCCGCACGGGAATGATTAGCGAGCCCAAGCTGGGAATGATCCTTATGGCCGTCCGCTTCGTTCGCGAAGGCTCGGTTCAAGATGTCACGCTCTTGCCCAGCAGCATCGACTACGAGCGAACGCTCGAAACCGAGGGTCACGTGGCGCAGCAGCTGGGCTCCAAAAAGAAAAAGGAGTCTCTCTTTGGCACCATCCGCTCCGGCCTTGGCATCCTTTTCGGCGCGTCCAACTATGGCCAGGCCTACGTAAACTTTGCAGACGGCATCTCTGTGCGCAAGGTCATCGACGAAGTCGAGGCTGCGCACGGCGGTGAACAGGACGTCTGCTATGTCCCCGAGGAAGTCTACGTCAAGGCTGTCGCCCGCCATGTGATCCTCGGTCAAAGGCGCGTCAGTTATCTTACCCCGGTTGCTTTCGTGGCCGCTGCGCTCCTTGGATCTCCCGAACACGAGAGCAACGAAGAACCTATGAGCAAGGGGAGCGCTAGCAGTCACATGTCTTCTTTCGCAGATGTCGACATCCTCGCGCACCCCGAGACCGGCCTGCTGCGACGCATGGCGTTTTTATTTCGCCTTGCTCGTGCCTCCGGTGCCTGCTTGCCGCCCAACTTGGATCTCGAAGATGACGATAATGCCATTGCAGTCATTGAACGCGCTCAGGAGGTGCTGCGTCCGTTTCTCGCACCCGTTTCCACTCATAACGAGCAAGTGCCCAAGACCGTCTCAAGCTGA |
| *ScACS* | A2978 | ATGGTCGCAGAGGCGGCGCTCTGCGCCGCTCTGCAAGGCGACGACGGCGGCGGCTCGGGCCGCGGCAATCGAGGCAAAGCAAACCCGAAACTCGCCAACGAGCAAGAGAACAAGCGCAAGAAGAAGAGCGAGCAAGAGCAGCAGCAGCAAGCAACCAGCCAGCCAGCCAGCCAGACAGACAAGATGGCCGAGGAGAAGGGCAACGCCGTGGGCGAGGTCGTCCACGATGTGCGCTCGCACTCGAGCAAGGGCGCCCACGTCGCCAGCCTCGAAAAGTACAAGAGGCTCCACGAGGACTCCGTCCGCGACCCCAGCGGATACTGGGGCCGCCTCGCCCGCCGCGAGCTCAAGTGGATGCACGACTTCGACAAGGTCAAGATCGGTTCCCTCCAGAACGGCGACCTCGCCTGGTTTGTCGGCGGCAAGCTGAACGTCTCGGAGAACTGCCTCGATCGCTGGGTTGAGGAGGGCCGCGGCGACGACGTCGCCATCATCTGGGAAAAGGATGAGCCCGGCCAAGCCGTCAAGTTCACCTACCTCGAGGTCCTCCACAAGGTGTGCCAGATCGCCAACGTCATGCGCAAGTTCGGCGTCAAGAAGGGCGACCCCGTCACGTTGTACATGCCCATGATCCCCGAGGCCGCCTTTGCCCTCCGCGCCCGTGTCGACGACGTCCACTCCAAGTGGATCTTCACCGCCGACGAGGGCCTCCGCGGCGGCCGCACCATCCCCCTCAAGAAGACCGTCGACGATGCCATCTCCTCGTGCGACTTTGTCAACAAGTGCTTTGTCTTCAAGCGCACCGGCGCTGATGTCAGCATGACCGAGGGCCGCGATCTCTGGATGGAGCCCGAGCTCGAGGGCCAGCGCCCCTACTGCCCGCCCGAGGTCATGGACGCCGAGGACCCGCTCTTCTACCTGTACACGTCCGGATCCACCGGCAAGCCCAAGGGTCTCATGCACTCCACCGCTGGCTACATTCTTTACGCCCAGACCACCTGCAAGATCATCTTCGATCTTCGCCCCGGCGATGTCTACGCCTGCGTCGCTGATGTCGGCTGGATTACCGGCCACAGCTACATTGTCTACGGCCCCCTCGCCAACGGCACCACCACGCTCATGTTCGAGTCCACCCCGCTCTACCCGGATGCCGGCCGCTACTGGGACGTCGTCGAGACCCACGCGGTGACCCAGTTCTACACGGCCCCCACCGCCATCCGCGCCCTCATGCGCCACGGCCCGGAGCCCACTGAGAAGTACGACCTCTCCACCCTCCGTATCCTCGGTACCGTCGGCGAGCCCATCAACCCGGCCGCCTGGACCTGGTACTTTAAGCACGTCGGCAAGGAGCGCTGCGCCATCATTGACACCTACTGGCAGACCGAGACCGGTGGTATCATTGTCACCCCGCTCCCCGGCGCCACCCCGCTCAAGCCCGGCTCCGCCACCCTTCCCTTCTTCGGCATTGACCTCGAGGTCCTCGACCCCCAGAGCGGCAAGGTCGTTCCCTTCGTTGAGGGCGAGGAGTCCTCGGGTGTGCTCTGTGTCAAGTCGCCCTGGCCTTCCATGACGCGCACCGTCTTCAACAACCACGAGCGCTACATGAACACCTACCTCAAGCCCTACCCCGGTTACTACTTTACCGGCGATGGCGTGACCCGCGATAAGGACGGCTTCTTTTTTGTCACTGGCCGTGTCGATGACGTGCTCAACGTCTCCGGCCACCGCATCGGCTCCGCTGAGATCGAATCTGCGCTCGTCGCCCACGACGCCGTCGCCGAGTCTGCATGCGTTGGCTTCCCCCACGAGGTCAAGGGCGAGGGTATTGCCTGCTACGTCATCCTCAAGCTCGGCAACGAGGAGACCGACGACCTCATCAAGGATCTCCGCAACCAGATCCGCTCCGTCATTGGCCCATTTGCCTCGCCGGACTACATTGTCATCACCCCGGCTCTCCCGAAGACCCGCTCGGGCAAGCTCATGCGCCGCGTGCTCCGCAAGATCATTGCGCTCGAGGCCGACCAGCTTGGCGACACCTCGACCCTCGCCGACCCCTCGGTCGTCGACAGCCTCATCGAGAAGGTTGCGACCCTCCGCAAGTAA |
| *ScACC* | A2520 | ATGATGCAGAACGAGAGCATCCCCGGCGCCGCGCAGGATGCCAAGCTCCGCACGTTCGAGAACATGGAGGAGTACGTCAAGTCGCAGGGCGGCAAGCGCGTAATCAAAAAGGTCCTCGCCGCCAACAACGGCATGGCCGTCGCCAAGCTTCTCAAATCCATCCGGTCCTTTTGCTACTCGACCTTTGGCCGCGAAAACGAGATCGAGGTCATCTGCATGGCCACCCCCGAGGATCTCGGCGCCAACGCCGAGTACATCCGTGCCGCCGATCAGGTCGTGCACGTCCCCGGAGGCTCGAACGTGAACAACTACAACAACGTCAGCCTCATCGTGGAGATCGCCCAGCAGTACAAGGTCGACGCCGTCTGGGCCGGCTGGGGTCACGCCAGTGAGAACCCGGTCCTCCCAGCCACTCTCTCCGAGCTCGGCATCGTTTTTGTGGGCCCCCCTGCCGGCCCGATGAATGCCCTCGGTGACAAGATCATGAGCAGCATCGTCGCGCAGTCGTGCGGCTGCCCCATGATCGCCTGGAACGGCTCCGACATCCGCGTCAACTACAAGGAAGATGGCGGTGTCTCGGACGAGATCTTCGACTCGGCCAACGTGCAGACCGTCGAGGACGCCAAGAAGCAGGTCGAGAAGATTGGCGTCCCCGTCATGATCAAGGCCAGTGAAGGTGGTGGCGGCAAGGGTATCCGCCTCGTCGACGACTACTCCAAGGTCGAAGCCTGCTTCCGTCAGGTCCAGAGCGAGGTGCCCGGCAGCCCCATCTTCATCATGCGCCTCGCCGAGCGCGCCCGCCATCTCGAGGTCCAGCTCCTCGCCGACGAGTACGGTAACGCCATTGCCCTCAGCGGCCGCGACTGCTCCGTCCAGCGTCGCCACCAAAAGATTCTCGAGGAGGGCCCGCCCGTGGCCGCCAAGCCCGAGGTCTGGAAGCAGATGGAGCACGCCGCCGTCAAGCTCGCCAAGGAGGTCGGCTACGTCAACGCCGGCACCGTCGAGTACCTGTACGATGACAACGACAACTTCTTCTTCCTGGAGCTCAACCCTCGTCTCCAGGTCGAGCACCCGGTGACCGAGATGATCACCGGCACCAACGTGCCGGCTGCCCAGCTCCAGGTTGCCATGGGCATCCCCCTCAACCGCATCCCGGACGTGCGCCGCTTCTACGGCTGCGACGATCTCTTTGCGGACGAGCCCATCGATTTTGACGGCGAGTACGCCGACGAGCCCACCACCACGAGCATTACTCGCGGCCACACCATTGCTGCCCGCATCACGGCCGAAAACCCCTTCAACGGCTTCCAGCCCACCATTGGCCAGATTAGCGAGATCAACTTCCGCAGCTACCGCAACGTGTGGGGCTACTTTAGCGTTGACAGCTACGGCCGCGTCCACGAGTTTGCCGACTCGCAGATTGGCCACGTCTTTGCCTGGGGCGAGACCCGCGAGGAGGCTCGCCGCAGCCTTGCCATGGCGCTCCACGACCTCTCGATCCGCGGTGAGATTCGCACCACCATCGAGTACCTCAAGGATCTCATCGAGTCCGAGGACTATGTCAACAACAAGTTTAATACCGCCTGGCTCGATGCCCGCATCAAGAGCAACATTGCCGTCTCCAAGATGGACCCGCTCACCATTGCTCTCGTCGGTGGTGTCTGCACCGCGCACCGCGAGATCGCCGCGCGTGGCGCTGACTACATGAGCATGCTTGAGCGTGGCCAGCTCCCGCCCGTGCCCCTGCTCGACCAGGCGCACGCCTTTGAGCTCATCTACGAGGGCGTCAAGTACAAGCTCAACGGCTGCCACACCGGCGAGAACACCTATCGCCTCTACTGCAACGGCAGCCACGTCGACGCCGAGCTCCGCTGCCTCGCGGACGGTGGCTTCCTCGTGCTCATTGGCGGCCGCTCTCACGTCGCCTACGTCAAGGAGGATGTTGGCGCCCAGCGCTACACTATCGACGGCCAGACCTGCCTCTTTGAGGACGAGTATGACCCGACCCAGATGCGCGCCCAGATGGGTGGCAAGCTCCTCCGCTACCTCGTCGAGGATGGTGCCTCGCTCGAGAAGGGCGACGGCTTTGCCGAGATTGAGGTCATGAAAATGAACATGACCCTCTCGGCCCTCGAGGCCGGTACCATCACGCTGCACAAGCCCGAGGGTGCCGTCATGGAGCCCGGTGACATGATTTGCACCATGGAGCTCAAGGACCCCAGCAAGGTGCAAAAGGCCAAGCTCTTCGAGGGCACCTTCCCCGCCCTTGGCGAGCCCTGGCCCCAGACTCTCCGCAACATGCCGCACCACACCCTCGAGCGCGCCCAGCGTCGCCTCGAGGCCGTCATGGCCGGTTTCGCGATCGAGAATGAGGTCACTGTCGAGGCCCTCAAGAGCCTCCGCGATGCCCTCCACAGCCCGCTCCTCCCCGTGCTTGAGATCGAGGGCATCGTCTCGCGCACCAAACACGCGCTTCCCAAAGCGCTCCTCAGCAAGGTCGAGAGCCTCTGCCGCGAGCTCCGCTCCAAGGAGGAGCCCAGCCTCGACGACTCGGCGGCCTTTGCCGCTGCCGTGCTCTCGGCGGCCCAGGAGCACCCTCCTGCTCAGGTTGGCGAGATCCTCTCGGTGGCCGAGTCGTACAAGGACGGCCTCAGTATCCTCTACGCGCGCATCCTTGGCAAGCTTATCGCCAGCTTTGTCGAGGTCGAGGCCAAGTTTGCCGAGCTCGAGAACTCGGACGCTGCCGACAAGGATGACGTCCTCCAGGAGCTCCGCGCCCAGAACGCTGGCGACCTCCCCAAGGTCCAGCGCTTTGCGCTCGCGCACCACTCGCGCAAGCCCCGCGACACCCTCGTGCTCGCCATCCTCGAGGAGCTCGACACCCTGCAGCGTGGCACCGAGACCCGCAGCGCCTCGCCCAAGCTTCGCTCCGAGTGTGCCAACATCATGCAGGAGGTCGCCGCTCTCCAGGGCATCAAGACCACGGATGTGGCCCTCGAGGCTCGCCAGTCGCTCATTGAGATGGAGCACTCGTACGAGGACCAGCTCAAGTCTGTAACTGACAAGCTCAAGCGCGTCATGCAGGGCGATGAGTCCGCCCGCGAGGAGCTCGTGCAGTCCACCGAGCCGGTTCTTGCTTACCTCATGGACATTGTCTCGCGCTTCAACGAGAACCCGGCCGACCTTCGCCAGACTGCCCTTCGCGTCTACGTGAGCCGCGTCTACGCTGCCTACAAGGTCGTTGATGCCTCGACGAGCGAGCTGACCAAGAACAACCTTGCCTGCGACTTTACGTTCTTCTCGGAGGCGACCGACTCTGTCCAGGTCGGCGGTGGCGGCTCGGGTCTCGCGAACGTGTCTTCGTTCGAGGACCTCACCAAGGTGCTCAGCAACAACGGCGACTTTGACTTTGGCGCGACCAACGAGTCGAACCGCTCGGATGCCGAGACCAGCAGCGTTGGCATGGACCTCGACATTGGCGGCGGCGCCGAGAGCAGCGCCCCGACCTCGGGCCTCTCTGGCGGCAAGTCGATGCTTGCGCGCCAGGGCGGCGACTTTAAGGCTCGCTCCGCCTCGGTGGAGGGCCCGGCCTCGTCCACGGTCCCGCCGTACGTGAACCGCAAGGGCAAGCTCCTCTTTTTCGCCGACATGGCCGAGCTCGAGGAGAACCTTGCGTCCTCGATCAAGGACTTTGGCTCCGACACTTCGTCGCCCACGCCGCTCAACGTCGTGCATGTCATCTTTGGCACGCTCGTCGACTCGGAGACTGATGTTTCGGCCAAGCTGTACGAGGTCGTGCAGAAGAACAAGGTTGTGCTCAGCGAGAACCTCGTGCGCCGCATCACCTTTGCCGTGGTGCGCTCCCAGTACGAGGAGGCTGACCGCCACGTGGCCATTGTTGCCGGCCATGGTGGCCACTTCTTCACTTTCCGCAACAGCTCCGGCTACGAGGAGGACCGTCTCGTGCGCAACATTGAGACGCCGCTGGCCTTCCAGCTCGACCTCGAGCGCATGTCGAACTTCAACATTCGCATGGTGCCCATCGGTCGCAGCATGAGCCGCTCGCAGGCTGTGCACGTGTACGAGGCGACGCCCAAGGCCAACGCCGCCGGCAAGGTTGTGGGCATGCGCCGCTTCTTTGTGCGCGCTCTCGTCCGCGATGCCGAGCGCGTCAAGCTCGACGTCGGCACCTTCGATGCCTACCCTGGCCCGGAGCGTGTCTTCGTGCAGGCCCTGCGCGCCCTCGAGTCCGTCCAGGACGACTCGAAGACCAAGGCCCGCAAGAACCACATCTTCATGAACGTGCTCAGCGACTCGGCCACCGTCGACGCTGGCTACGTCGAGGGCATCATCCGCACGCTCCACCGCCGCTACGCCAAGCGCCTGATTTCGCAGAACGTCGAGGAGTTTGAGATCCGCGTCAACGCTGTTCTCGCCGAGGGCGCGCCGAGCATGCCCATTCGCGTCATCGCCTCGAACCCGACCGGCTTTGCCCTCAACATCGACACCTACGTCGAGGCCTCGGACCCTTCGGGCTCGCAGGATGCCATGTACTACTCGATCTCCGAGGGCGACGGCGGCCTTGGCGGCGCCCTTGCCGCCATGGGCCTCGGTGCCGGTGAGGCCGACGTCAACGCCGGCTCCACGGACAGCACGGGCTCGCTCCACGGCAAGCCCCTGCACACGCCGTACCCGGTCTCGGACGAGTTCGACGAGCGCCGTGCCCGCGCCCGCGCTGCCAGCACGACCTTTGCGTACGACTTTCCGGATCTCTTCCGCAAGTCGCTCGAGTTTGCCTGGCGCGAGCACCTCAGCACGACCGGCACCAAGGAGAACATGCCGCCCCGCAAGAGCCTCGTCGAGGCCGAGGAGCTCGTCCTCGACGACGAGTTCGAGGCTGGCGATGCCGTCGACCCGGTCAACGCCCCCCGCCTCTGCCGCGTGGACCGCAAGGCCGGCCGCAACCCGATCGGCATGGTCGCGTGGCGCTTCTTCATGCGCACCCCGCAGTACCCGCGTGGCCGCGAGGTCGTGGTCATTGCCAACGACATCACCGTCAAGGCTGGCTCCTTTGGTACCCGCGAGGACATGCTCTTTGACCAGGCCTCCAAGTACGCGCGCCTGAATGGGCTCCCCCGCCTGTACATTGCCGCGAACTCTGGCGCCCGCATCGGCATGGCCGACGAGGTCAAGCGTGCCTTCCAGGTCAAGTGGATTAACGAGGCCGACCCGACCAAGGGCTACGAGTACATTTACGTGAACGAGGATACCTTCAATCAGCTCGGCCCCGACGGCCGCAAGAGCTTGCTCGCTGAGAAGGTCGAGGGCACGGACCACTTCCGCATCAACGCCATCGTTGGTGAGTCCCCGGACCTTGGTGTGGAGAACCTCCGCGGCTCGGGCACCATTGCCGGCGAGACGGCGCGTGCCTACGAGGAGTCCTTTACCCTCTCGTACGTGTCGGGCCGCTCGGTCGGTATTGGCGCCTACCTCGTCCGCCTTGGCCAGCGCATTGTGCAAAAGGGCAAGAACGCCCCGATCCTGCTCACCGGCTACCAGGCGCTCAACTCGCTCATGGGCCGCGAGGTGTACACCTCGAACTTGCAGCTCGGTGGCACCAAGGTGATGTTCGCCAACGGTGTCTCGCACCAGAGCGTCCGCCACGACCTCGAGGGTGTGGCCTCCATGGTCAAGTGGCTCTCGTACGTGCCGGAGCGCCGCGGCGCCCCGCTCCCGCTCACGGCCCTGGTCTCGGGTGACCGCATCGACCGCGACGTCGAGGTGCACCCGCGCGACCTCGGCTCGGACTACGACCCGCGCACCCTGCTCACCGGTCTCTCCAAGGAGGACGGCAGCTTCCTCGGCGGCTTCTTTGACAAGGACTCGTTTACCGAGACGCTCTCCGGCTGGGCTCGCACCGTCATTGCCGGCCGCGCTCGCCTGGGCAAGCTGCCCATGGGTGTCATCATCTCGGAGATTCGCACCGTCGAGGCGCGCGCGCCCGCTGACCCTGCGGCGCCCGAGTCCCAGGAGCTCATCTGGAACCAGGCCGGTCAGGTGTGGTTCCCGGACTCCTCGTACAAGACGGCGCAGGCCATCAACGACTTCAACCGCGAGGGTCTCCCGCTCATGCTCTTTGCCAACTGGCGTGGCTTCTCGGGCGGTACCCGCGACATGTTCGACCAGATTGTCAAGTTTGGTGCCTACATTGTCGACGCCCTCGTCGCGTACAAGCAGCCTGTCTTCGTGTACATCCCGCCCTTTGGCGAGCTGCGCGGCGGCGCTTGGGTCGTGGTCGACGAGACCATCAACCCGTCCATGATGGAGATGTACGCCGACACCGACGCGCGCGGTGGTGTCCTCGAGCCGGCCGGTGTCGTCTCGATCAAGTACCGTGCCAAGGATGTCCTCGCCACGGCGCACCGCGTCGACGAGAAGCTCAAGGGCATGGTCGAGAAGCTCAAGGCCGCCGCCCCGGACTCTGCCGAGGCCGCGGACCTGAAGAAGGCCATTGCCGAGCGCGAGAAGCTCCTCATGCCCATCTTCAAGCAGATTGCCGTGCACTTTGGCGACCTCCACGATCGCCCGGGCCGCATGCAGGCCAAGGGTGTCATCCGCAGCGTGGTCCCCTGGAGCAACTCGCGCCGCCACTTTTACAACCGCCTGCGCCGCCGTCTTGCCGAGCTCGACGCCGTCGCCAAGATCGACGAGGTCGTTGCCCTTTCGGACGACCTCGGTGCCCCGAGCTCGCAGCCGCTCGAGATTCTCGAGAGCGTCTTCAACGCCTCGTGCGACTCGAGCGTCCCGGACTGGAACAACGATAAGACGGTCCACGAGTGGCTTGTCTCCGAGCAGGGCCAGCAGGCCGTGTCCAAGCACCTCGCCGGCATCAAGGCGGACGCTATCTCGAACAAGGTCACTTCCCTCGGCATGGAGGACCCCAAGGCCATCCTCAAGGGCCTCATGGGCGTCATCTCTAAGCTCCGCGACGAGGAGCGTGAGGAGGAGCGCGCCGCTCTCGTCAACCTTCTGCGCAAGGGCTCGCTCCTGCTCAATTAA |
| *ScPDC* | A8969 | ATGGCCTTCGCGATGCGCGGCGCGCTGGGCAGCCTGGGGCGCGCGGCGACGTCGATGGGCATGGCGCAGACGCAGACGCAGGCGGCGCTGGGCGCGGGCCGCGGCAGCGGCGCCGCCGCGGCGCAGAGGCTCGCGGGCATGGCGCGCGCGATGAGCTCGCTCCCGAGCCACACCAAGCTCACGCTCCCCGCGCTCTCGCCCACCATGAGCGAGGGCGTCATCGCGAGCTGGCTCAGGAAAGAGGGCGACTTCGTGGAGGCCGGCCAGCCCGTCTGCGAGATCGAGACCGACAAGGCCACCGTCGACTTTGAGGTCCAGGACGACGGATACCTCGCGCGCATCCTCGTCGAGGCCGGCGGGCCCGCCCAGCCCGTCGGTGAGGTCATCGGCGTCCTCGTCGAGGACAAGGAGGACGTCGCCGCCTTTGCCAATGTGACCCTTGCCGACATGTCCGAGGGCGGATCCGCCCCGGCCCCGGAGAAGAAGGAAGAGCCCGCACCCGCCAAGCAAGAGCAGAAGAAGGAAGAGCCCGCCCCTGCGCAGACCTCTGCCCCCGCTGCGTCCTCCACCCCCGCTGGCCAGTCCTCGGGCGAGCGCGTCTTTGCGAGCCCGCTCGCGCGCAAGCTCATGACCGAGAACAACGTCGACCTCTACGCGCTCACCGGAACCGGACCCAACGGCCGCATCATCAAGGCTGACGTCGAAGACTACCTCGCATCCCCGCAGGCCCAGGAGGCCGCCCCCGCCGAGGCCGCCTCCTCCGAGGCCCCTGCCGCCTTGAGCGGCGCCTCGGGCGACTACTTTGACGTTCCTGTGGCCGAGGACGCCCAGGAACTCGCTGCCCAGCTCGAGCAGAGCAAGCACGAGGTGCCCCACTACTACCTCAACTGCGACATCGATGTCAGTGCCCTGCTCGATGTGCGCGCCCAGCTCAACGCCAAGCTCCCCGAGGACCAGCAGGTGTCCGTCAACGACTTTGTCGTGCGCGCCGCTGCTCTCTCCATGCAGGCCGTTCCTGACGTGAACGCCAGCTGGGGCAAGACCTTTATCCGCCAGTACCGCACCGTCGACATCAACGTCGCCGCTCTCACCTCGCCGGACGATGGCGTGCTCATGCCTGTGATCCGCGGCGCCGAGCGCAAGGGTCTCACCGCCATCTCCGCCGAGATGCGCTCCTTCGTCTCGGACAGCGATGACGTTGACCTTGACCTCGGTGTCGGCACCTTTACCATCTCCAACATGGGTGCCTTTGGCGTCAAGACCTTCACCCCGATCGTGCGCCAGCCCCAGGGCTCCAGCCTCGGCGTCGGCACCATCACCCGCCGTGCTGTCCCTGTCGCTGCCTCTATCGAGGCCGACCTTTCCCAGGGCCCCCCTGTGAAGATTGTCCCCACCATGACCGTCACGCTCTCCAGCGACCACCGCGTCGTGGACGGCGCCGTCGGCGCGCGCTGGCTCCAGCACTTCAAGACGCTCCTGGAGTCCCCGCTCAACATGCTTCTCTAA |
| *ScG6PD* | A6492 | ATGTTGGACCAAATGATGAGCGAGGAGGAGTACGCTCCCGCAGAGTTCCTCGACCCGGACCAGGAGACTTGGCATGAGGAGAGGCTGACCTTGGTCGTCTTTGGAGCCTCGGGAGACCTGGCCAAGAAGAAGACGTTCCCGGCCATCTTTGACCTCTTTCGCGTCGGCGCCCTCCCGCGACTGACCATTGTGTGCGGCTATGCAAGGTCCAAGATGAGCGACGAGGATTTTCGCTCGCGTATCCGTGAATACCTCGAGGGAAAGGGCTCCGAGGAGGAGCTCGAGCGCTTCCTCGACATTTGCATCTACCGCAGCGGACAGTACGACGGCGTCGAGGAATTCAAGGAGACAAGCGCCGAGCTCCGCGCCCTCCACGAGTGCAAGGACGACCAGGAGGTAGAGAATCGCATGTACTACCTGGCCATCCCTCCCAACCAGTTTCTCAACACGATCAAGACTACTCGCGAGGGAGGTATGTCCAAAACCGGATGGACTCGCGTCGTTGTGGAGAAGCCCTTTGGCCAAGACACCGAATCGGCGAAAAAGCTTGGCGACGACATTAGCCAGTATTTTGACGAGTCCCACCTGTACCGCATCGATCACTACCTGGGCAAGGAGATGGTGCAGAACCTCGTCAGTCTTCGCTTCGGCAACGCCTTCCTTGAGCCTCTATTTAATCGTGACCACGTCAAATCCGTCATTATCTCCTTCAAGGAGCCCTTTGGCACAGAGGGCCGCGGCGGCTACTTTGACTCGTATGGAATTATCCGCGACGTCATGCAAAACCATCTTATGCAGCTTCTCAGCATCATCGCAATGGAGCCGCCCGTAAAGGTTGCCGGCACCACCAAAGACGGAACCGACTATTCGCGCTTTGTTCGCGATGAAAAGGTCAAGGTCCTTGCTGCTGTCAAACCCTGGTCCCTCGATGACGTCGTTCTCGGCCAGTACGTCTCCAACGGGGACAAGCCGGGCTACCTCGACGATGACACCGTCCCCGAGGGTTCCAACCAGCCCACCTACGCGGCTGTGCGCATGTTCATTCATAACAAGCGCTGGGATGGCGTGCCTTTTATCATGAAGGCTGGCAAGGCGCTCGACGAGAAAAAGTGCGAGGTGCGTTTCCAGTTCCGTGACCCGGCCGGCGCAGCCGCCATGTTCGGCGAGTCGCGCATTCCGCGTAACGAGCTTGTACTCCGCCTCCAGCCCCAGGAGAGCATCTACATGAAGCTCAACGTGAAGAAGCCCGGTCTCGCCACTACCATGGTGCAGAGTTCGCTCGACCTCGACTACACAGATCGCTACGACGATCACCAGATTCCGGAGGCCTACACGCGTCTTTTGCTCGACGTTTTGCGTGGAAAGCAAGCAACCTTTGTTCGCGACGACGAGCTTCTTGCTGCCTGGGAAATTGTCACGCCTCTTTTGGAGGAAATCGAGTCGGGTGATGTCAAGCCCATTCCGTACGAGTACGGATCGCGTGGACCTGCCGAGGCTGACGAGCTCGTTAAGGACTCTGGATACGTTCGCAACGCCGAGTACGCAGCACGCTACAACGACTGGAAGGAGTCCAAGAAGAAGTAA |

**Additional file 1: Table S2.**Strains and plasmids used in this study.

| **Name** | **Description** | **Source** |
| --- | --- | --- |
| **Strains** |  |  |
| E. coli DH5α | supE44 ∆lacU169 (φ80 lacZ ∆M15) hsdR17 recA1 endA1 gyrA96 thi-1 relA1 | Ktsm-life |
| PO1f | matA, URA3-302, LEU2-270, xpr2-322, axp2-delta NU49, XPR2::SUC2 | ATCC |
| CK | PO1f Δ*DGA1* Δ*DGA2* Δ*LRO1*::HisG-URA3-HisG | This work |
| yL-EPA | PO1f-Δ12-Δ9-Δ8-Δ5-Δ17::HisG-URA3-HisG, P_TEFin_-Δ12-T_xpr2t_, P_pgpd_-Δ9-T_mig1t_, P_EXP_-Δ8-T_lip2t_, P_TEFin_-Δ5-T_CYC1T_, P_EXP_-Δ17-T_xpr2t_ | This work |
| ScDGAT2A | CK-ScDGAT2A::HisG-URA3-HisG, P_TEFin_-ScDGAT2A-T_CYC1T_ | This work |
| ScDGAT2B | CK-ScDGAT2B::HisG-URA3-HisG, P_TEFin_- ScDGAT2B-T_CYC1T_ | This work |
| ScDGAT2C | CK-ScDGAT2C::HisG-URA3-HisG, P_TEFin_- ScDGAT2C-T_CYC1T_ | This work |
| ScDGAT3 | CK-ScDGAT3::HisG-URA3-HisG, P_TEFin_- ScDGAT3-T_CYC1T_ | This work |
| ScDGAT2AM | ScDGAT2A embedding *ScDGAT2A* mutant H596Y/S598P, HisG-URA3-HisG | This work |
| ScDGAT2BM | ScDGAT2B embedding *ScDGAT2B* mutant F606Y/G608P, HisG-URA3-HisG | This work |
| yl-EPA::ScDGAT2C | yl-EPA-ScDGAT2C::HisG-URA3-HisG, P_TEFin_- ScDGAT2C-T_CYC1T_ | This work |
| yl-EPA::ScDGAT2AM | yl-EPA-ScDGAT2AM::HisG-URA3-HisG, P_TEFin_- ScDGAT2AM-T_CYC1T_ | This work |
| yl-EPA::ScDGAT2BM | yl-EPA-ScDGAT2BM::HisG-URA3-HisG, P_TEFin_- ScDGAT2BM-T_CYC1T_ | This work |
| yl-EPA::ScDGAT2C  ::ScGPAT1 | yl-EPA::ScDGAT2C-ScGPAT1::HisG-URA3-HisG, P_TEFin_-ScGPAT1-T_CYC1T_ | This work |
| yl-EPA::ScDGAT2C  ::ScGPAT2 (yL-EPA-1) | yl-EPA::ScDGAT2C-ScGPAT2::HisG-URA3-HisG, P_TEFin_-ScGPAT2-T_CYC1T_ | This work |
| yL-EPA-2 | yl-EPA-1-ScG6PD::HisG-URA3-HisG, P_TEFin_-ScG6PD-T_CYC1T_ | This work |
| yL-EPA-3 | yl-EPA-1-ScACS::HisG-URA3-HisG, P_TEFin_-ScACS-T_CYC1T_ | This work |
| yL-EPA-4 | yl-EPA-1-ScACC::HisG-URA3-HisG, P_EXP_-ScACC-T_xpr2t_ | This work |
| yL-EPA-5 | yl-EPA-1-ScPDC::HisG-URA3-HisG, P_TEFin_-ScPDC-T_CYC1T_ | This work |
| yL-EPA-6 | yl-EPA-2-ScPDC::HisG-URA3-HisG, P_TEFin_-ScPDC-T_CYC1T_ | This work |
| yL-EPA-7 | yl-EPA-3-ScACC::HisG-URA3-HisG, P_EXP_-ScACC-T_xpr2t_ | This work |
| yL-EPA-8 | yl-EPA-6-ScACC-ScACS::HisG-URA3-HisG, P_EXP_-ScACC-T_xpr2t_, P_TEFin_-ScACS-T_CYC1T_ | This work |
| **Plasmids** |  |  |
| pUC57 | ColE1 Ori, LacZ, Amp | GenScript |
| pUC-HUH | HisG-URA3-HisG in pUC57 | This work |
| pUC-intF3-HUH | intF3 upstream and downstream homology arms inserted into pUC-HUH | This work |
| pUC-intF3-ScDGAT2A | P_TEFin_-*ScDGAT2A*-T_CYC1t_ cassette in pUC-intF3-HUH | This work |
| pUC-intF3-ScDGAT2B | P_TEFin_-*ScDGAT2B*-T_CYC1t_ cassette in pUC-intF3-HUH | This work |
| pUC-intF3-ScDGAT2C | P_TEFin_-*ScDGAT2C*-T_CYC1t_ cassette in pUC-intF3-HUH | This work |
| pUC-intF3-ScDGAT3 | P_TEFin_-*ScDGAT3*-T_CYC1t_ cassette in pUC-intF3-HUH | This work |
| pUC-intF3-ScDGAT2AM | P_TEFin_-*ScDGAT2AM*-T_CYC1t_ cassette in pUC-intF3-HUH | This work |
| pUC-intF3-ScDGAT2BM | P_TEFin_-*ScDGAT2BM*-T_CYC1t_ cassette in pUC-intF3-HUH | This work |
| pUC-HUH-ScGPAT1 | P_TEFin_-*ScGPAT1*-T_CYC1t_ cassette in pUC-HUH | This work |
| pUC-HUH-ScGPAT2 | P_TEFin_-*ScGPAT1*-T_CYC1t_ cassette in pUC-HUH | This work |
| pUC-HUH-ScACC | P_EXP_-*ScGPAT1*-T_Xpr2t_ cassette in pUC-HUH | This work |
| pUC-HUH-ScACS | P_TEFin_-*ScACS*-T_CYC1t_ cassette in pUC-HUH | This work |
| pUC-HUH-ScPDC | P_TEFin_-*ScPDC*-T_CYC1t_ cassette in pUC-HUH | This work |
| pUC-HUH-ScG6PD | P_TEFin_-*ScG6PD*-T_CYC1t_ cassette in pUC-HUH | This work |

**Additional file 1: Table S3.** Primers used in this study.

| Primers | Sequence (5’ > 3’) |
| --- | --- |
| ScDGAT2A-F | GAGTATAAGAATCATTCAAAATGCTAGCGCGCAAGGTGGG |
| ScDGAT2A-R | TGACATAACTAATTACATGACTAAAAGGTGGGGAGTGTGA |
| ScDGAT2A-1 | TTTAGCCAAGGGTATAAAAG |
| ScDGAT2A-2 | AAAATGCAGTCTTGCTCGGC |
| ScDGAT2A-3 | AATGGCTGGGCATTCTTCCA |
| ScDGAT2A-4 | ATTGTTGAAGACCATTTCAG |
| ScDGAT2A-5 | TTTTGGGGGATCCAATCGAG |
| ScDGAT3-F | GAGTATAAGAATCATTCAAAATGACCCTGACCGGGCCGGA |
| ScDGAT3-R | TGACATAACTAATTACATGATCACAGTGTCAACATTGACG |
| ScDGAT3-1 | TTTAGCCAAGGGTATAAAAG |
| ScDGAT3-2 | AGATCAGTATGCGGAATGAT |
| ScDGAT3-3 | AATTGCTTGTATCCTAACAT |
| ScDGAT2B-F | GAGTATAAGAATCATTCAAAATGGAGGCCGCCAAGAACGA |
| ScDGAT2B-R | TGACATAACTAATTACATGATCAATTGTCAATGAAAGAAA |
| ScDGAT2B-1 | TTTAGCCAAGGGTATAAAAG |
| ScDGAT2B-2 | ATAATAATGGTATGCTCGCG |
| ScDGAT2B-3 | AAATCTTTGGATGGTCCTTT |
| ScDGAT2B-4 | TCTACATTTCCACGACCTTT |
| ScDGAT2B-5 | AATCTTCATGCTCGGAAAGT |
| ScDGAT2C-F | GAGTATAAGAATCATTCAAAATGCAGACACCGTACAGCAC |
| ScDGAT2C-R | TGACATAACTAATTACATGATTACTTTTGAATGAGCAGCG |
| ScDGAT2C-1 | TTTAGCCAAGGGTATAAAAG |
| ScDGAT2C-2 | TTCTGCTTCTCTTCCCGCTC |
| ScDGAT2C-3 | ACACCAATTTTCAGTGGCCG |
| ScDGAT2AM-F | TTGTTGAAGACTATTTCCCGCTCTCGATGGTGCGCACGTC |
| ScDGAT2AM-R | CGGGAAATAGTCTTCAACAATGCGAAAAAGGAATGATCCG |
| ScDGAT2BM-F | TCGCGGAAAAGTATTTCCCGTTGCGTCTCCAGCTCACCGA |
| ScDGAT2BM-R | TGGAGACGCAACGGGAAATACTTTTCCGCGAGGCGAGGAA |
| ScDGAT2AM-4 | ATTTATCTCACAACCTATCT |
| ScDGAT2BM-2 | ATACATGTCGCAACGCAGAC |
| ScDGAT2BM-3 | TTTCTCAGTTTTGTGCTCTA |
| ScDGAT2BM-4 | ATAATCTCATTCGAATGGGC |
| ScGPAT1-F | GAGTATAAGAATCATTCAAAATGAGCGACCACGACAACAA |
| ScGPAT1-R | TGACATAACTAATTACATGACTACATGGAGCTTGTCTTGC |
| ScGPAT1-1 | TTTAGCCAAGGGTATAAAAG |
| ScGPAT1-2 | AAGACGACAATGCTGGCTTT |
| ScGPAT1-3 | ATTGTAATCTTCGTGCTTGG |
| ScGPAT1-4 | AAGAAGTCCATTGCCGACAA |
| ScGPAT2-F | GAGTATAAGAATCATTCAAAATGCTCCGGCAAGGGTCCGC |
| ScGPAT2-R | TGACATAACTAATTACATGATCAGCTTGAGACGGTCTTGG |
| ScGPAT2-1 | TTTAGCCAAGGGTATAAAAG |
| ScGPAT2-2 | TCAACATTCCCGTCATCGGA |
| ScGPAT2-3 | ACATGTCTTCTTTCGCAGAT |
| ScG6PD-F | GAGTATAAGAATCATTCAAAATGTTGGACCAAATGATGAG |
| ScG6PD-R | TGACATAACTAATTACATGATTACTTCTTCTTGGACTCCT |
| ScG6PD-1 | TTTAGCCAAGGGTATAAAAG |
| ScG6PD-2 | AGTATTTTGACGAGTCCCAC |
| ScG6PD-3 | ATCTACATGAAGCTCAACGT |
| ScACS-F | GAGTATAAGAATCATTCAAAATGGTCGCAGAGGCGGCGCT |
| ScACS-R | TGACATAACTAATTACATGATTACTTGCGGAGGGTCGCAA |
| ScACS-1 | TTTAGCCAAGGGTATAAAAG |
| ScACS-2 | TCAAGTTCACCTACCTCGAG |
| ScACS-3 | TACTGGGACGTCGTCGAGAC |
| ScACS-4 | TGAGATCGAATCTGCGCTCG |
| ScPDC-F | GAGTATAAGAATCATTCAAAATGGCCTTCGCGATGCGCGG |
| ScPDC-R | TGACATAACTAATTACATGATTAGAGAAGCATGTTGAGCG |
| ScPDC-1 | TTTAGCCAAGGGTATAAAAG |
| ScPDC-2 | AAGCAAGAGCAGAAGAAGGA |
| ScPDC-3 | TTTACCATCTCCAACATGGG |
| TEF-F | GAGTATAAGAATCATTCAAA |
| CYC1t-R | TGACATAACTAATTACATGA |
| ScACC-F | CACAAGACATATCTACAGCAATGATGCAGAACGAGAGCAT |
| ScACC-R | ACAAGTTCCGTAGTTGGATCTTAGTTGAGCAGGAGCGAGC |
| EXP-F | CACAAGACATATCTACAGCA |
| Xpr2t-R | ACAAGTTCCGTAGTTGGATC |
| ScACC-1 | ATTATATATAAGGCTCGTCT |
| ScACC-2 | AAGATCATGAGCAGCATCGT |
| ScACC-3 | GACAACGACAACTTCTTCTT |
| ScACC-4 | CCCGCATCAAGAGCAACATT |
| ScACC-5 | AAAAGGCCAAGCTCTTCGAG |
| ScACC-6 | TTTGCGCTCGCGCACCACTC |
| ScACC-7 | TTTGACTTTGGCGCGACCAA |
| ScACC-8 | AACTTCAACATTCGCATGGT |
| ScACC-9 | TCAACGCCGGCTCCACGGAC |
| ScACC-10 | AAGGGCTACGAGTACATTTA |
| ScACC-11 | TTCTTTGACAAGGACTCGTT |
| ScACC-12 | TGAAGAAGGCCATTGCCGAG |


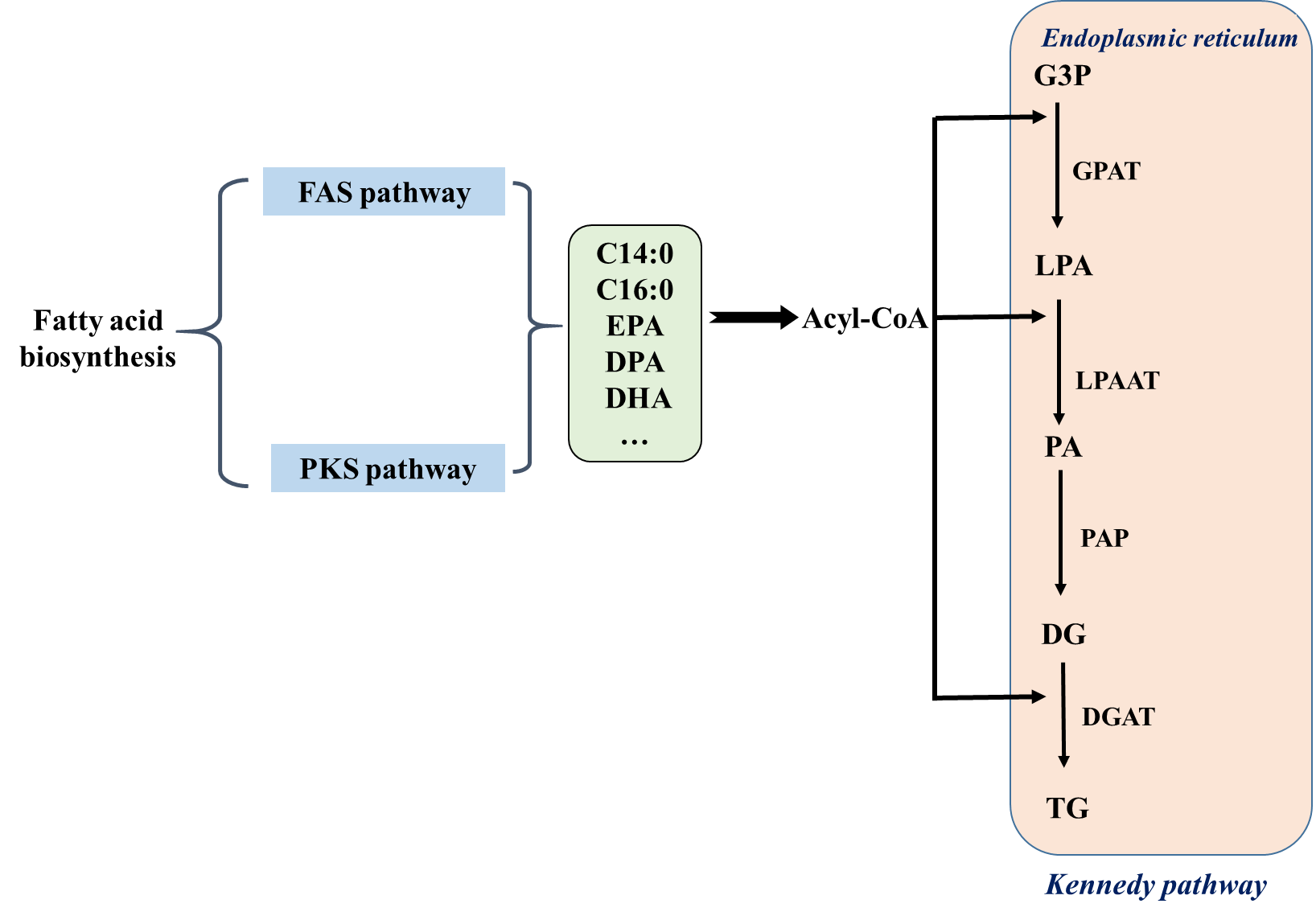


**Additional file 1: Fig. S1.** Biosynthesis pathway of triglyceride in *Schizochytrium* sp. HX-308. FAS: fatty acid synthase; PKS: polyketide synthase; EPA: eicosapentaenoic acid; DPA: docosapentenoic acid; DHA: docosahexaenoic acid; G3P: glycerol-3-phosphate; GPAT: glycerol-3-phosphate acyltransferase; LPA: lysophosphatidate; LPAAT: lysophosphatidate acyltransferase; PA: phosphatidate; PAP: phosphatidic acid phosphatase; DG: diacylglycerol; DGAT: diacylglycerol acyltransferase; TG: triacylglycerol.


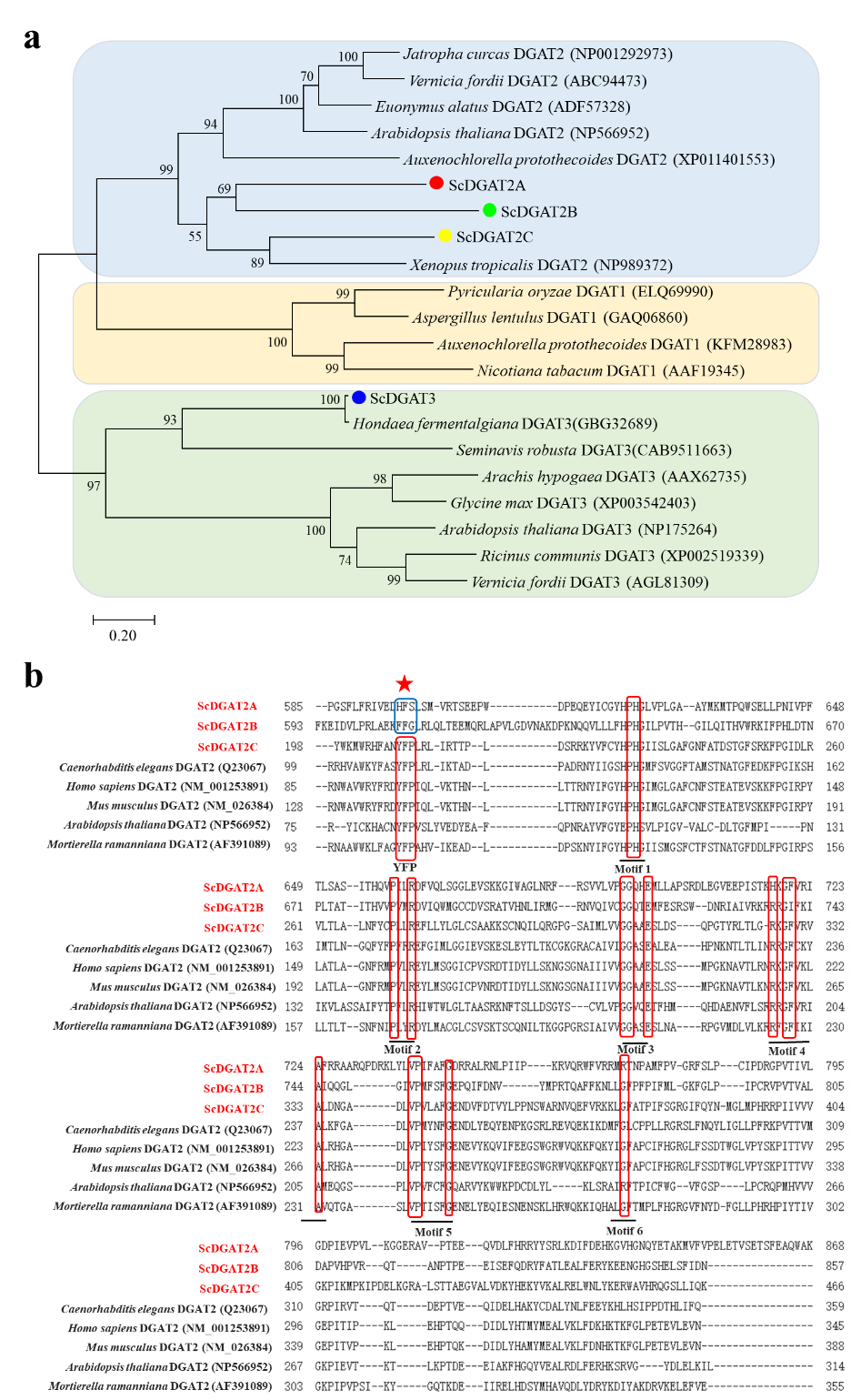


**Additional file 1: Fig. S2.** Amino acid sequence analysis of four *DGAT*s. (a) Phylogenetic analysis of amino acid sequences of *ScDGAT2A*, *ScDGAT2B*, *ScDGAT2C*, and *ScDGAT3*. The neighbor-joining method was used to reconstruct the cladogram under the software MEGA 7. The scale bar 0.2 represents 20% divergence. The bracket after the species name represents the GenBank ID. (b) Protein sequence alignment of *ScDGAT2A*, *ScDGAT2B*, and *ScDGAT2C* with *DGAT2*s from five organisms.


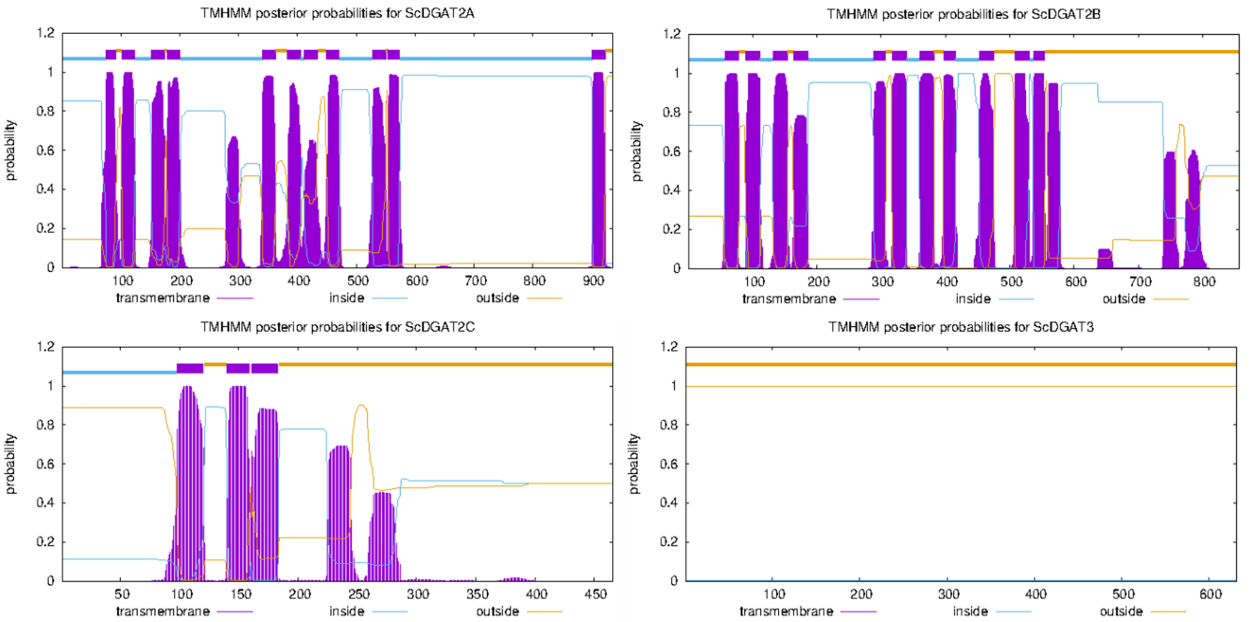


**Additional file 1: Fig. S3.** Predicated transmembrane domains for *DGAT*s by TMHMM.


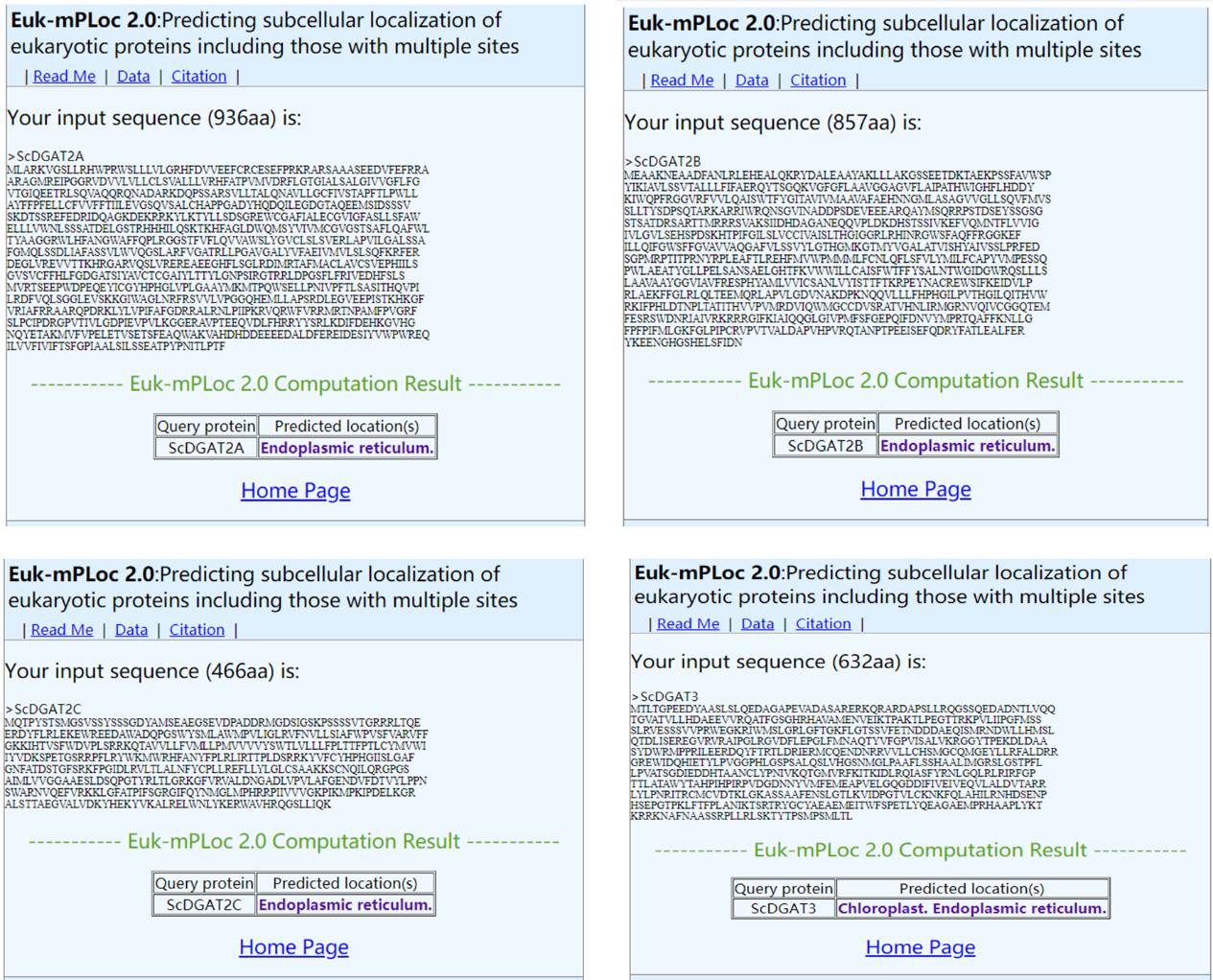


**Additional file 1: Fig. S4.** Predicated subcellular localization of proteins for *DGAT*s.


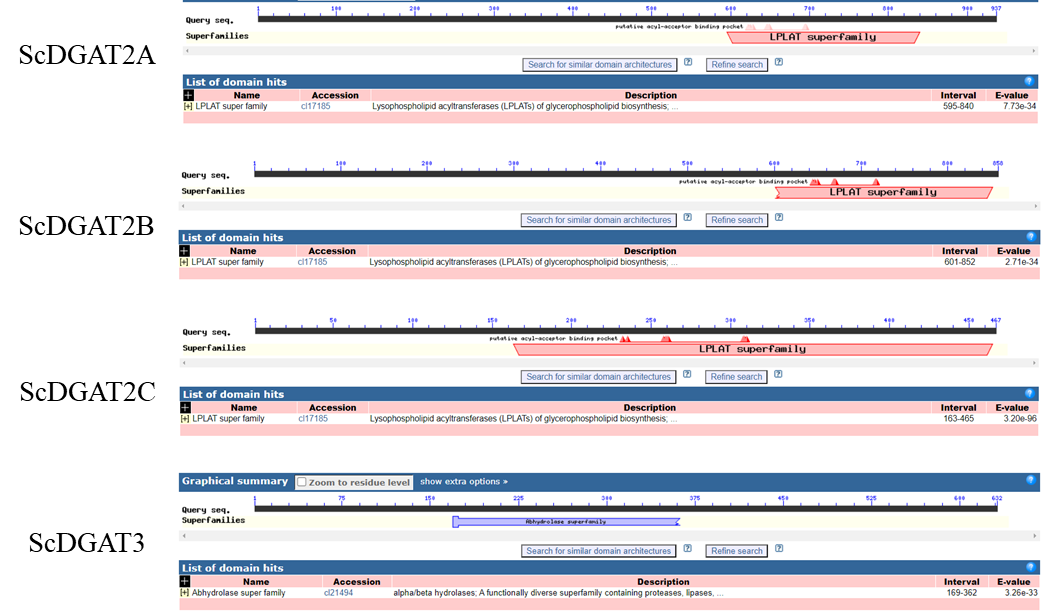


**Additional file 1: Fig. S5.** Conserved domains detected in *DGAT*s by NCBI Conserved Domains Search.


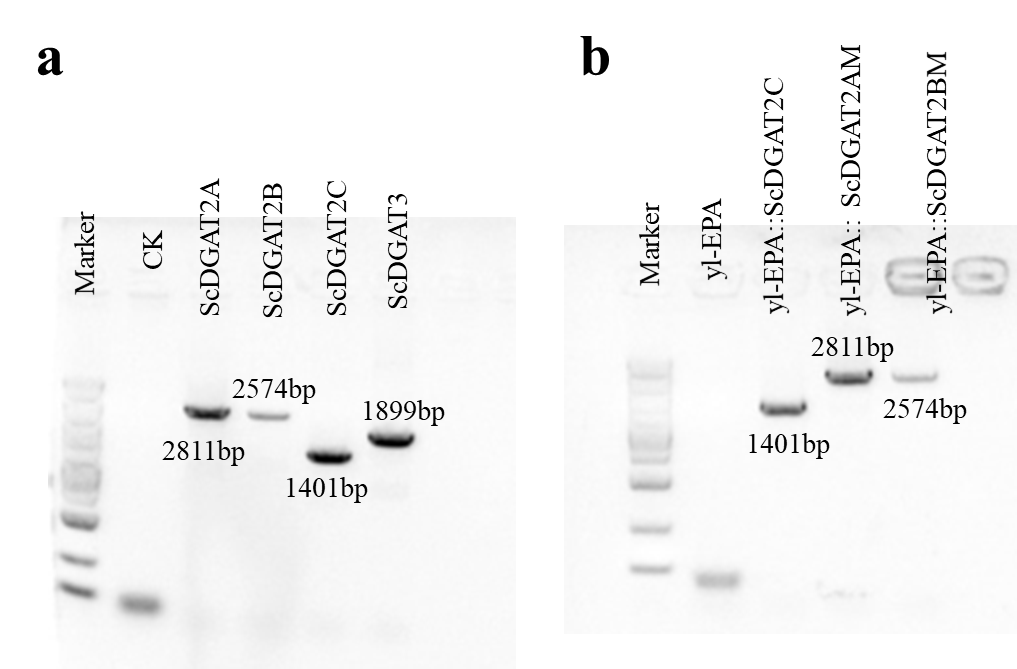


**Additional file 1: Fig. S6.** PCR validation of *DGAT* genes integration into CK strain (a) and yl-EPA strain (b) genome.


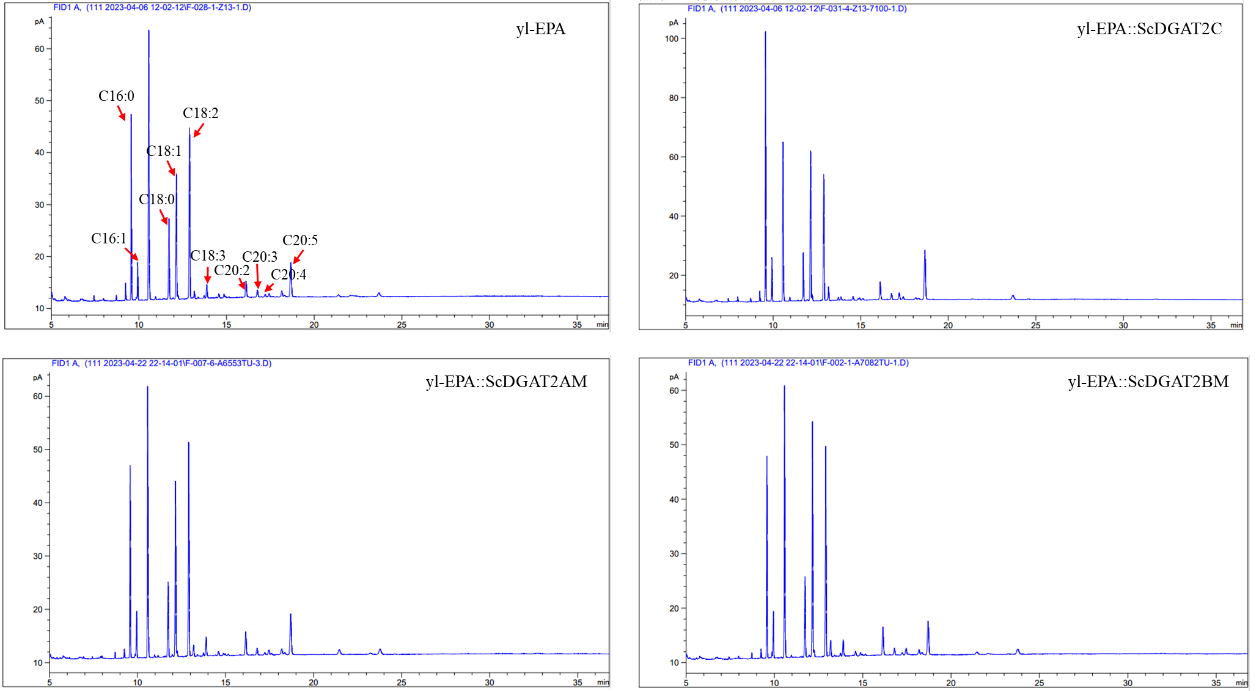


**Additional file 1: Fig. S7.** Gas chromatography (GC) analysis of *ScDGAT2C*, *ScDGAT2AM*, and *ScDGAT2BM* expressed sample.


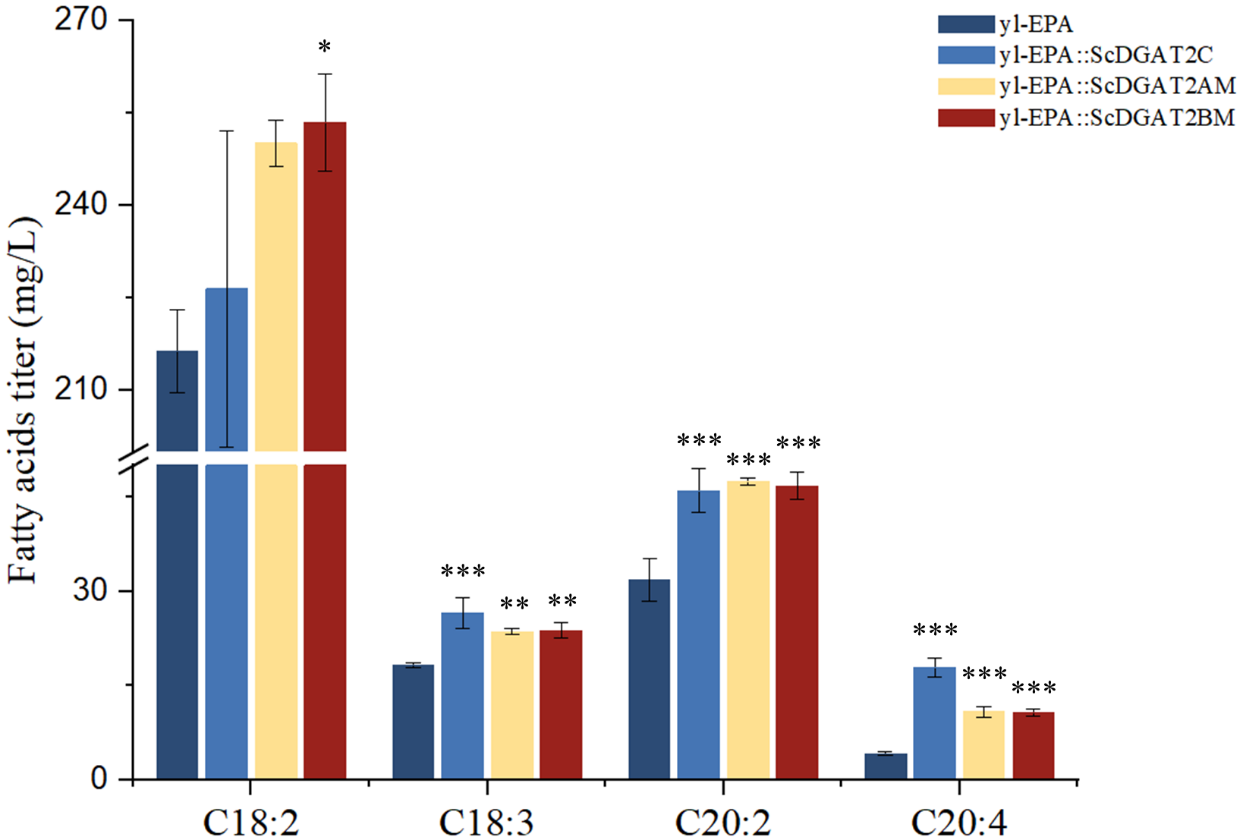


**Additional file 1: Fig. S8.** Effect of *DGAT*s expression on fatty acids titer. Three biological replicates were used and mean values ± SD (n=3) are shown. Two-way ANOVA with Tukey’s multiple comparisons test. *P < 0.05, **P < 0.01, and ***p < 0.001 compared with the yl-EPA-1.


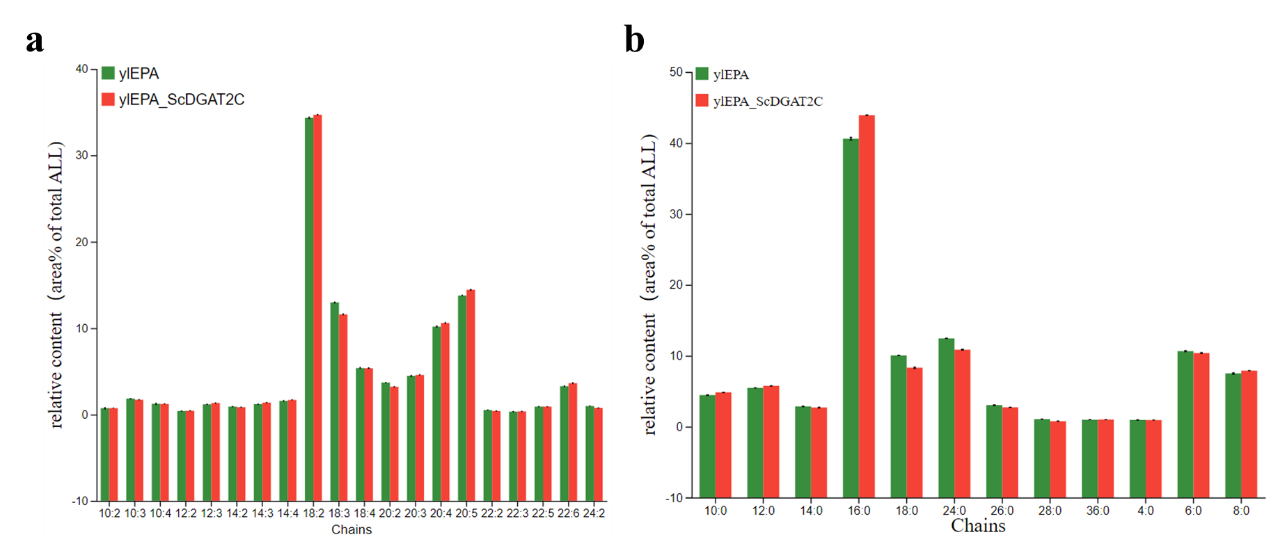


**Additional file 1: Fig. S9.** Effect of *ScDGAT2C* expression on PUFAs (a) and SFAs (b) composition in yl-EPA strain.


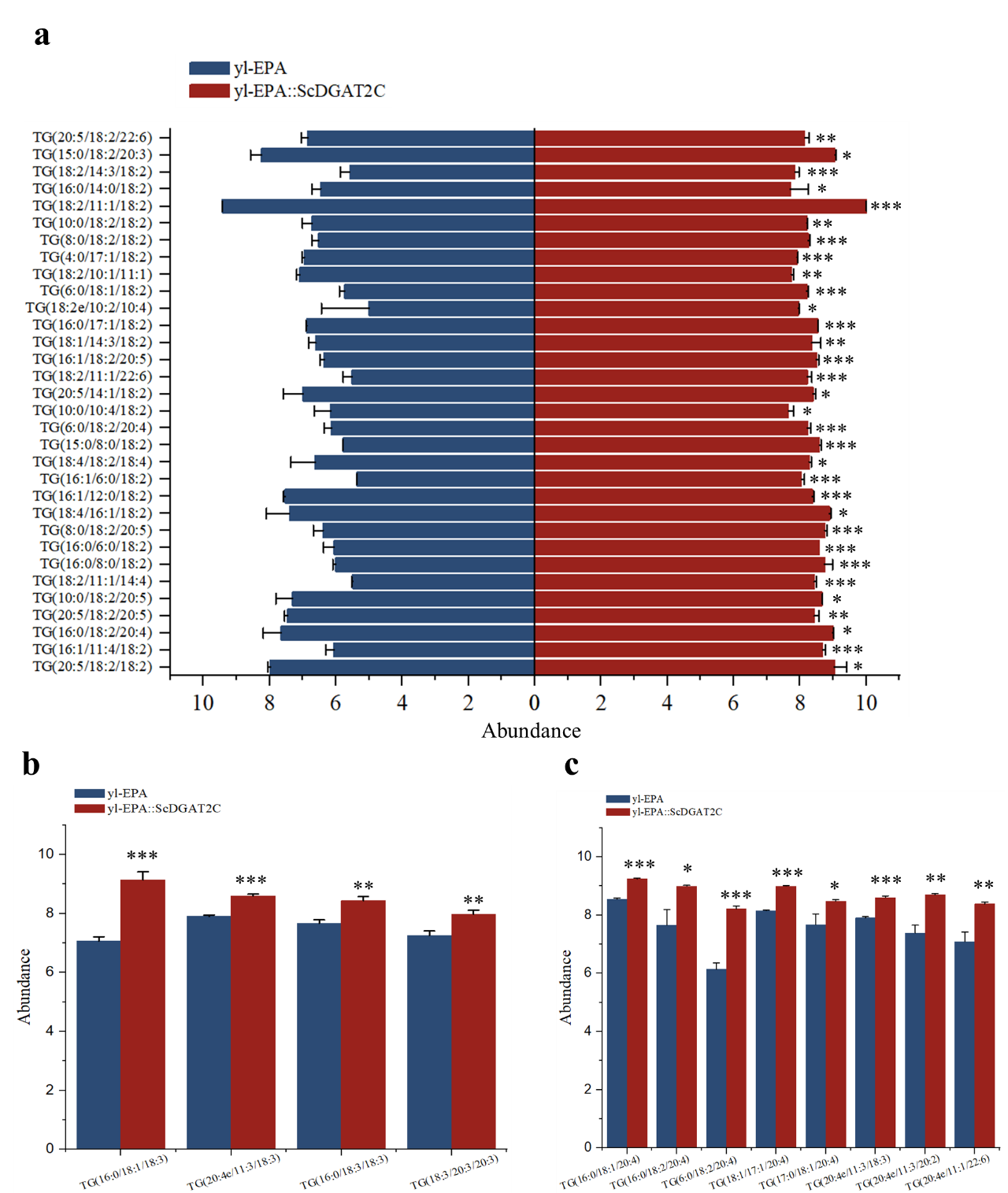


**Additional file 1: Fig. S10.** Effect of *ScDGAT2C* expression on fatty acid composition in TG. Effects of Sc*DGAT*2C on TG associated with C18:2 (a), 18:3 (b), and 20:4 (c) in yl-EPA strain. Three biological replicates were used and mean values ± SD (n=3) are shown. Student’s t test was used for statistical analysis, and statistical significance is indicated as *P < 0.05, **P < 0.01, and ***p < 0.001.


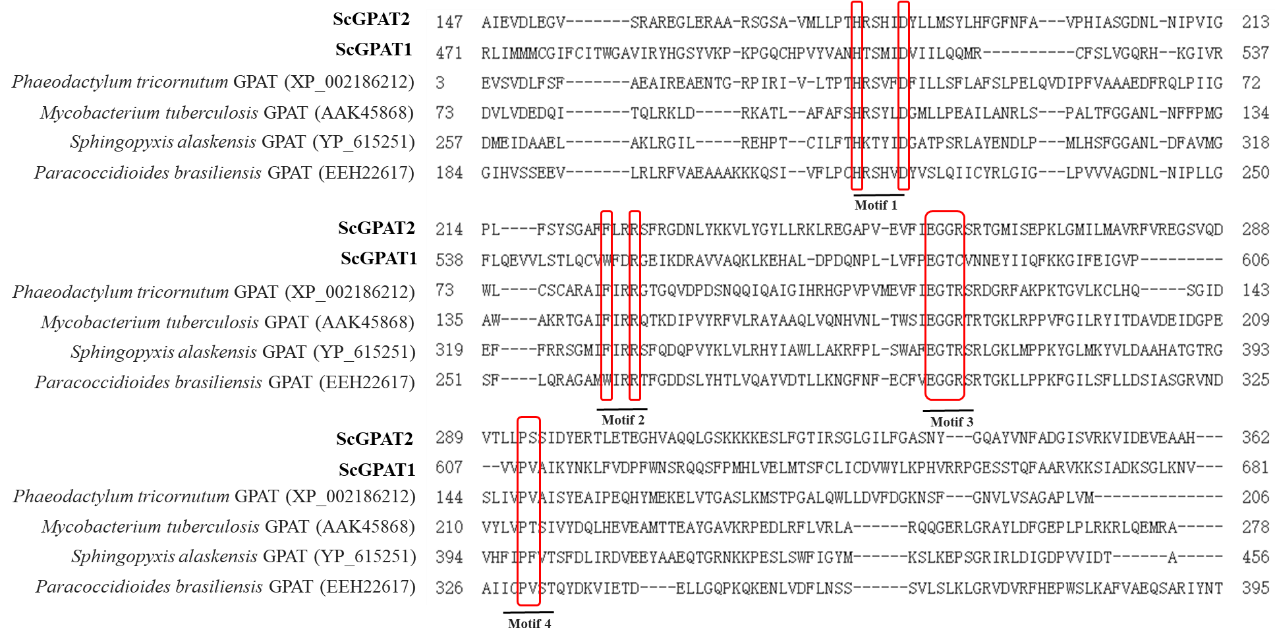


**Additional file 1: Fig. S11.** Protein sequence alignment of *ScGPAT1* and *ScGPAT2* with *GPAT*s from five organisms.


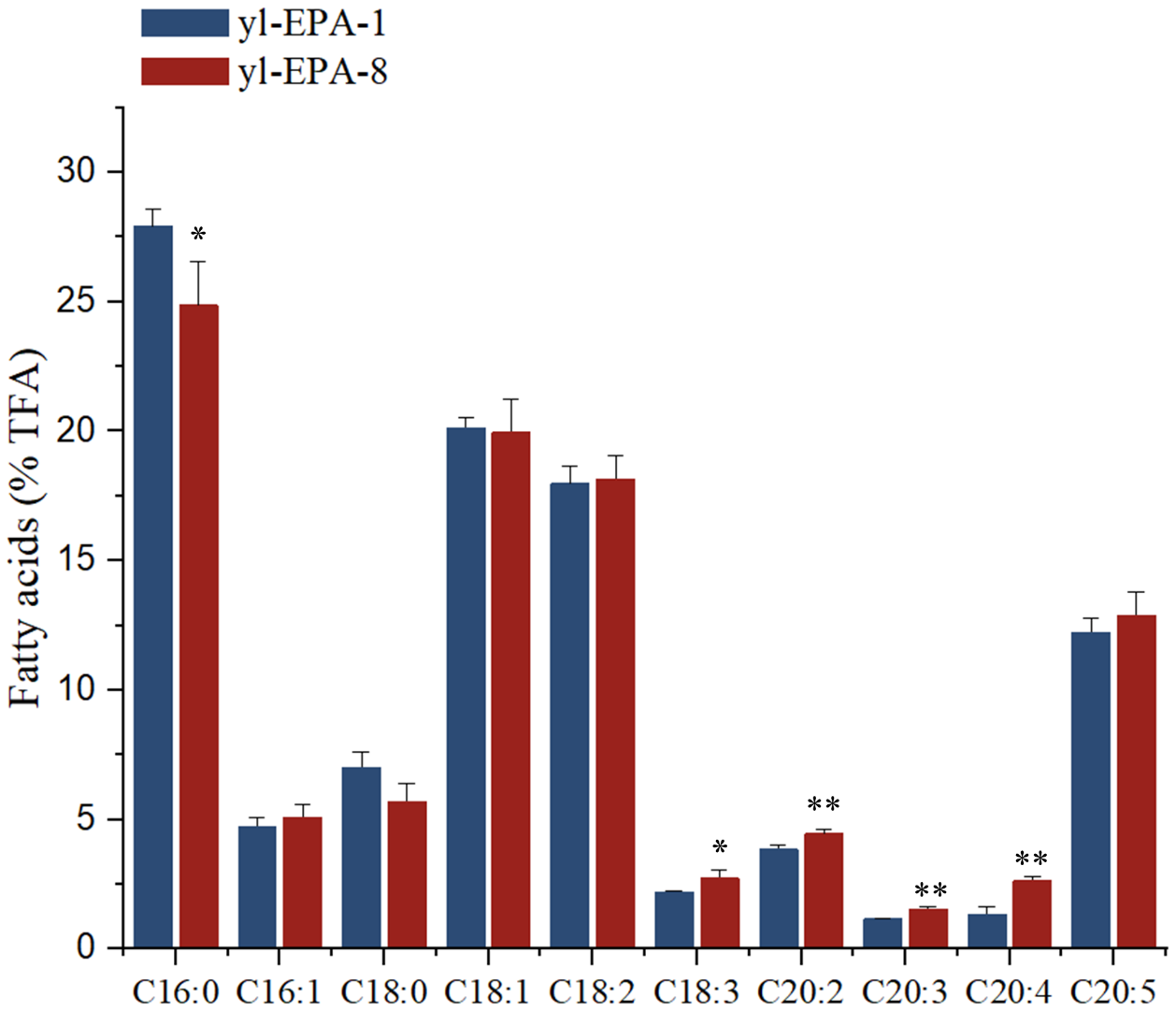


**Additional file 1: Fig. S12.** Comparison of fatty acid composition between yl-EPA-1 and yl-EPA-8 strains. TFA: total fatty acids. Three biological replicates were used and mean values ± SD (n=3) are shown. Student’s t test was used for statistical analysis, and statistical significance is indicated as *P < 0.05, **P < 0.01, and ***p < 0.001.
